# Supplementary material for: Estimating Health Adjusted Age at Death (HAAD)
Source: PLoS One. 2020 Jul 14;15(7):e0235955. doi: 10.1371/journal.pone.0235955 (PMC7360045; doi:10.1371/journal.pone.0235955)
Supplement: S1 Appendix — (DOCX) [file pone.0235955.s001.docx]

**Appendix**

**A1 Incidence**

Figure A1 shows details on incidence assumption that is being used in HAAD calculations.

[
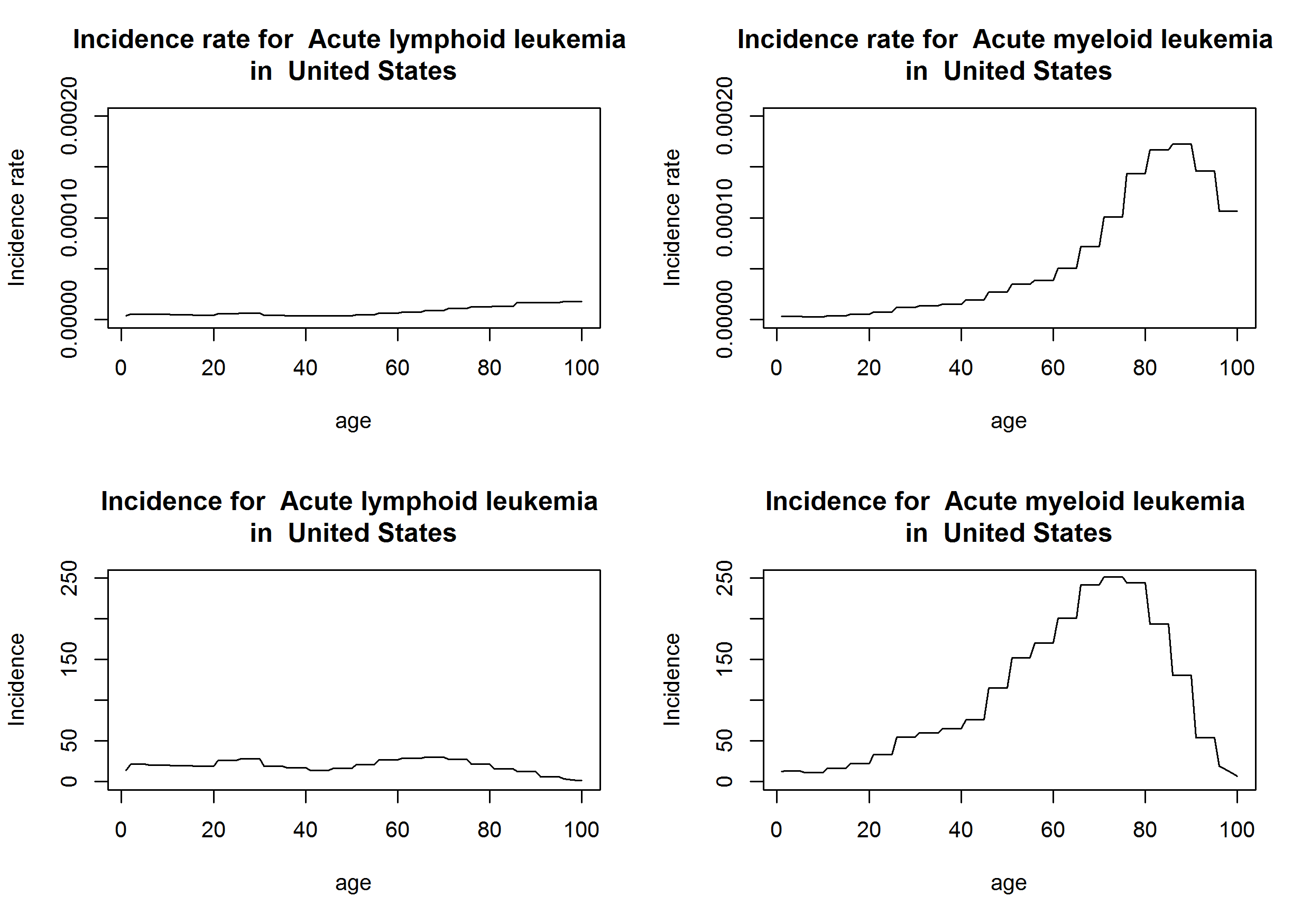


**Figure A1:** Incidence rates (top) and incidence (bottom) across age groups for AML and ALL in the United States (2017) [20].

**A2 Decomposition**

To illustrate how differences in demography, epidemiology, and disease specific mortality and morbidity led to differences in average HAAD (aHAAD) between Japan and Ethiopia as exemplars, we took an approach that parallels methods described by Das Gupta [33], creating estimates using the 64 possible scenarios of the two values for the 6 input factors. For each factor, there are then 32 pairs of scenarios in which all five other factors remain constant but the factor in question takes on either Japan or Ethiopia’s value. To calculate the effect of each factor on the overall HAAD difference between Ethiopia and Japan, we took the average of the difference in estimated HAAD between these 32 sets of paired scenarios. Essentially, this is the average effect of varying that one factor with every possible combination of other factors. Figure A2 shows the results of this decomposition. As expected, the younger age structure in Ethiopia and the higher overall mortality contributes substantially to differences in HAAD. However, we also see that disease-specific mortality differences play a large role for epilepsy, leading to a much lower HAAD in Ethiopia. The disease-specific disability plays a very small role for schizophrenia, as discussed in the main text, because of a lack of variation in the distribution of severity across countries in the input data from the GBD data (see A3). Surprisingly, the age distribution of incidence contributes to higher HAAD in Ethiopia, a function of the age distribution of incidence rates from GBD, which tend to be higher at older ages and lower at younger ages in Ethiopia.


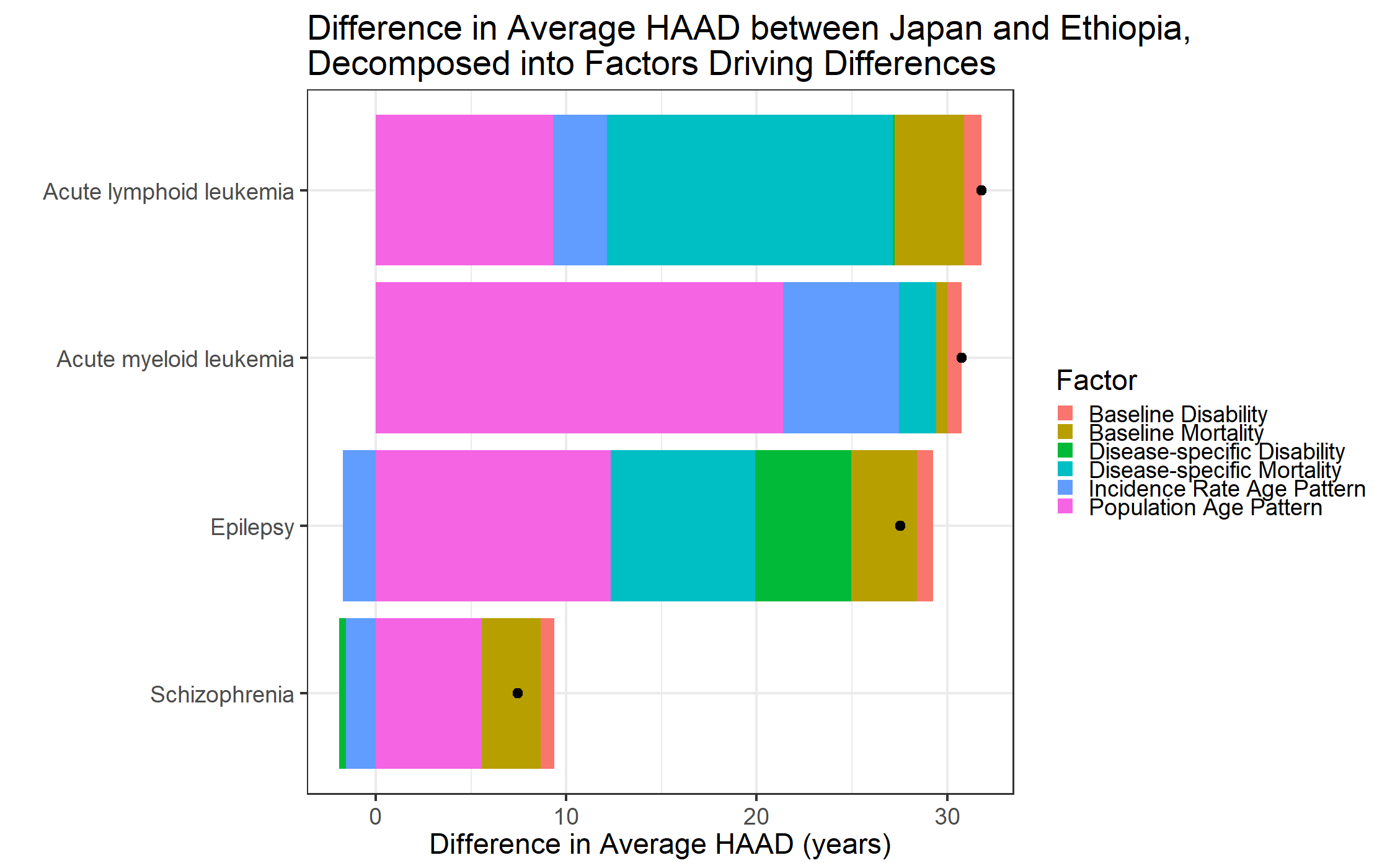


**Fig A2:** Difference in HAAD between Japan and Ethiopia for 4 diseases (Acute lymphoid leukemia, Acute myeloid leukemia, Epilepsy and schizophrenia), decomposed into factors driving differences.

**A3 Age-standardized average disability weight**

Mental disorders like major depressive disorder (MDD), bipolar disorder, and schizophrenia show almost no difference in age-standardized average disability weight between high income and low income countries in GBD, despite higher treatment rates in high income settings. We might expect the distribution of severity to be more severe in low income settings where treatment is less available. Differences in estimated HAAD between high and low income settings, particularly for nonfatal conditions like mental health disorders, are limited by these estimated severities in GBD. Note the much larger difference in average disability weight for epilepsy.


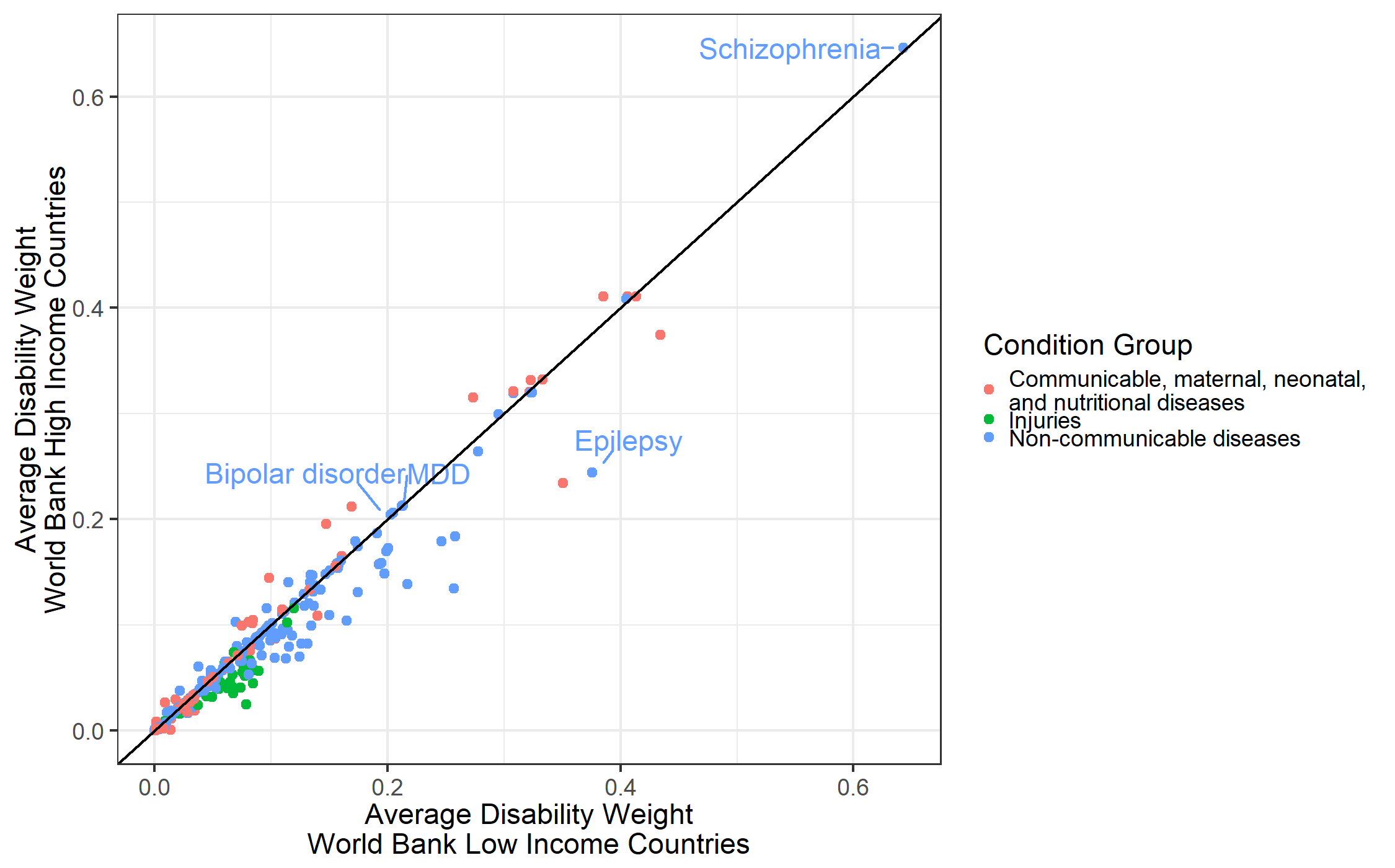


**Figure A3:** Age-standardized average disability weight (YLDs/Prevalence) in High Income versus Low Income countries by World Bank income groups.

MDD=Major depressive disorder

**A4 HAAD for 200 diseases in 6 countries**

Table A1 shows estimated average HAAD (aHAAD) for 200 NCDI conditions for Ethiopia, Haiti, China, Mexico, US and Japan.

**Table A1:** aHAAD for 200 NCDI conditions for Ethiopia, Haiti, China, Mexico, US and Japan (T20 is the % with HAAD<20, Q1 is quartile 1 and Q3 is quartile 3).

|  | Ethiopia | | | | Haiti | | | | China | | | | Mexico | | | | US | | | | Japan | | | |
| --- | --- | --- | --- | --- | --- | --- | --- | --- | --- | --- | --- | --- | --- | --- | --- | --- | --- | --- | --- | --- | --- | --- | --- | --- |
|  | aHAAD | T20 (%) | Q1 | Q3 | aHAAD | T20 (%) | Q1 | Q3 | aHAAD | T20 (%) | Q1 | Q3 | aHAAD | T20 (%) | Q1 | Q3 | aHAAD | T20 (%) | Q1 | Q3 | aHAAD | T20 (%) | Q1 | Q3 |
| Acne vulgaris | 63.6 | 0.6 | 58.2 | 71.7 | 60.3 | 0.7 | 53.4 | 69 | 69.2 | 0.1 | 64.3 | 75.5 | 67.5 | 0.4 | 61.5 | 75.7 | 67.3 | 0.2 | 62.1 | 74.6 | 73.3 | 0.1 | 69.4 | 79.3 |
| Acute glomerulonephritis | 61.5 | 4.6 | 56.7 | 71.7 | 57 | 6.5 | 50.2 | 69 | 61 | 2.9 | 52 | 72.8 | 68 | 1.3 | 63.1 | 76.5 | 69.4 | 0.1 | 65.1 | 75.3 | 74.6 | 0.1 | 71 | 80 |
| Acute lymphoid leukemia | 34.8 | 44.8 | 8.6 | 62.6 | 35.1 | 41.8 | 9.7 | 60.2 | 58.3 | 5 | 48.4 | 72.3 | 46 | 22.3 | 21.4 | 68.3 | 58.4 | 4.6 | 50.4 | 72.2 | 66.6 | 1.5 | 61.4 | 77.2 |
| Acute myeloid leukemia | 32.7 | 38.7 | 8.7 | 54.4 | 35.1 | 30.1 | 14.9 | 52.8 | 48 | 14.3 | 33.2 | 64.3 | 41.1 | 21.5 | 22.6 | 59.1 | 59.3 | 2 | 53.4 | 68.2 | 63.5 | 1.5 | 58.1 | 72.9 |
| Adverse effects of medical treatment | 59.6 | 6.2 | 54.2 | 71 | 55.7 | 7.3 | 49.3 | 68.3 | 70.8 | 0.1 | 65.9 | 76.2 | 67.4 | 2 | 62.3 | 75.7 | 70.6 | 0 | 66.4 | 75.8 | 74.5 | 0.2 | 70.9 | 79.9 |
| Age-related and other hearing loss | 66.2 | 0.1 | 61.4 | 73 | 64.3 | 0.1 | 59 | 71.1 | 71.5 | 0 | 67.5 | 76.9 | 71.1 | 0 | 66.9 | 77.5 | 71.2 | 0 | 67.7 | 76.3 | 76.4 | 0 | 73.5 | 81.1 |
| Age-related macular degeneration | 69.3 | 0 | 65.5 | 73.7 | 68.2 | 0 | 64.3 | 72.6 | 73.1 | 0 | 69.6 | 77.3 | 73.4 | 0 | 70 | 77.7 | 72.9 | 0 | 69.9 | 77 | 77 | 0 | 74.4 | 80.8 |
| Alcohol use disorders | 60.1 | 0.1 | 54.4 | 67.3 | 57.1 | 0.1 | 50.6 | 64.7 | 66.2 | 0 | 61.5 | 72.3 | 62.5 | 0.1 | 56.1 | 70.7 | 62.6 | 0 | 56.9 | 70.2 | 68.5 | 0 | 63.6 | 75.2 |
| Alcoholic cardiomyopathy | 54.5 | 3.9 | 42.2 | 67.7 | 42.7 | 15.9 | 27 | 57.8 | 60.5 | 1.5 | 55.2 | 69.5 | 59.8 | 2.7 | 51.3 | 71.4 | 62.4 | 1.4 | 56.5 | 71.9 | 71.4 | 0.7 | 68 | 77.6 |
| Alopecia areata | 63.3 | 0.3 | 57.7 | 70.9 | 60.6 | 0.3 | 54.5 | 68.5 | 69 | 0 | 64.3 | 74.9 | 67.7 | 0.1 | 62.5 | 75.2 | 67.8 | 0 | 63.6 | 74.2 | 73.3 | 0 | 69.9 | 78.9 |
| Alzheimer's disease and other dementias | 70.4 | 0 | 67.1 | 74.5 | 68.9 | 0 | 65.2 | 73.3 | 72.9 | 0 | 69.6 | 77 | 73.7 | 0 | 70.5 | 77.8 | 72.6 | 0 | 69.5 | 76.5 | 76.8 | 0 | 74.2 | 80.4 |
| Amphetamine use disorders | 56 | 0.2 | 50.4 | 63.6 | 54.4 | 0.2 | 48 | 62.2 | 62 | 0.1 | 57.4 | 68.5 | 59.8 | 0.4 | 53.8 | 67.9 | 57.5 | 0.3 | 49.4 | 66.8 | 67.6 | 0 | 63.7 | 73.4 |
| Anorexia nervosa | 58.2 | 0.6 | 52.1 | 66.4 | 55.3 | 0.7 | 48.4 | 64 | 63.9 | 0.1 | 58.9 | 70.9 | 62.2 | 0.5 | 56.2 | 70.7 | 61.8 | 0.3 | 56.7 | 69.5 | 67.3 | 0.2 | 63.4 | 74.5 |
| Anxiety disorders | 63.7 | 0.6 | 57.9 | 72 | 60.9 | 0.5 | 54.7 | 68.7 | 69.6 | 0.1 | 64.8 | 75.8 | 68.2 | 0.2 | 62.8 | 76 | 67.8 | 0.1 | 62.6 | 74.8 | 73.7 | 0 | 70.5 | 79.6 |
| Appendicitis | 61.9 | 3.6 | 56.5 | 71.5 | 56.2 | 6.4 | 49.1 | 68.2 | 69.6 | 0.2 | 64.9 | 76 | 66.5 | 1.6 | 60.5 | 76.2 | 67.4 | 0.4 | 62.6 | 74.5 | 73.2 | 0.1 | 69.3 | 79.1 |
| Asbestosis | 58 | 0.2 | 52.6 | 64.1 | 57.6 | 2 | 51.5 | 65.5 | 67.9 | 0.1 | 63.9 | 73.6 | 63.2 | 0.1 | 57.9 | 70 | 68.3 | 0 | 64.9 | 72.4 | 71.2 | 0 | 67.3 | 75.5 |
| Asthma | 59.3 | 1.8 | 53.2 | 68.3 | 56.2 | 2 | 49.3 | 65.4 | 67.4 | 0.4 | 62.9 | 73.8 | 65.2 | 0.8 | 59.8 | 73.6 | 66.1 | 0.4 | 61.9 | 73.3 | 72.7 | 0.1 | 69.2 | 78.4 |
| Atopic dermatitis | 63.1 | 1.6 | 57.4 | 71.6 | 60.2 | 1.6 | 53.3 | 68.9 | 69.8 | 0.2 | 65 | 76.1 | 68.1 | 0.5 | 62.2 | 76.3 | 68.4 | 0.2 | 64.2 | 75.1 | 74.3 | 0.1 | 70.8 | 79.9 |
| Atrial fibrillation and flutter | 68.4 | 0 | 64.5 | 73.2 | 65.5 | 0 | 61.1 | 70.9 | 71.8 | 0 | 68.2 | 76.1 | 70.9 | 0 | 67 | 76.1 | 71.3 | 0 | 68.2 | 75.5 | 73.8 | 0 | 70.7 | 78.1 |
| Attention-deficit/hyperactivity disorder | 62.3 | 1.7 | 56.9 | 71 | 59 | 1.9 | 52.9 | 68.4 | 68 | 0.5 | 63 | 74.8 | 66.4 | 0.9 | 60.9 | 75 | 66.2 | 0.5 | 61.5 | 73.9 | 72.3 | 0.2 | 68 | 78.6 |
| Autism spectrum disorders | 50.7 | 7.4 | 46.8 | 60.5 | 47.7 | 8.4 | 42.7 | 58.2 | 57.8 | 1.6 | 54 | 64.2 | 56.3 | 2.8 | 51.6 | 64.5 | 56.6 | 1.5 | 52.2 | 63.7 | 62 | 0.6 | 58.4 | 67.6 |
| Benign and in situ cervical and uterine neoplasms | 64.5 | 0.3 | 58.3 | 71.8 | 62.2 | 0.1 | 56 | 69.8 | 69.9 | 0 | 64.4 | 75.6 | 69.3 | 0 | 63.2 | 76.5 | 67.7 | 0 | 62.2 | 74.7 | 73.5 | 0 | 69.5 | 79.4 |
| Benign and in situ intestinal neoplasms | 67.3 | 0.2 | 62.3 | 73.2 | 63.2 | 0.9 | 57.6 | 70.5 | 71.7 | 0 | 67.6 | 76.3 | 70.6 | 0.3 | 66.3 | 77.2 | 70.3 | 0 | 65.8 | 75.3 | 75.4 | 0 | 71.7 | 80.1 |
| Benign prostatic hyperplasia | 68.1 | 0 | 63.4 | 73.6 | 65.7 | 0 | 60.7 | 71.4 | 72.3 | 0 | 68.3 | 77 | 71.1 | 0 | 66.7 | 76.9 | 70.8 | 0 | 67 | 75.7 | 75.2 | 0 | 71.8 | 80 |
| Bipolar disorder | 55.9 | 0.6 | 50.9 | 62.6 | 53.7 | 0.7 | 48.1 | 60.8 | 64.3 | 0.1 | 59.7 | 70 | 60.2 | 0.4 | 55.2 | 67 | 61.2 | 0.2 | 56.6 | 67.4 | 67.1 | 0.1 | 62.9 | 72.8 |
| Bladder cancer | 64.6 | 0.1 | 59.6 | 71 | 63.5 | 0 | 58.1 | 69.7 | 70.6 | 0 | 66.4 | 75.9 | 69.8 | 0 | 65.2 | 76.1 | 70.6 | 0 | 66.3 | 75.6 | 75.7 | 0 | 72.3 | 80.4 |
| Brain and nervous system cancer | 37.3 | 35.7 | 10.7 | 60.7 | 40.8 | 25 | 19 | 59 | 63 | 2.2 | 56.8 | 73 | 51.2 | 12.2 | 38.6 | 67 | 61.5 | 1.5 | 54.4 | 71.1 | 72.2 | 0.6 | 68.3 | 79.1 |
| Breast cancer | 60.5 | 0.1 | 51.4 | 70.1 | 59.7 | 0 | 51.8 | 68 | 69.3 | 0 | 64.1 | 75.3 | 68.4 | 0 | 62.1 | 76.2 | 70 | 0 | 65.6 | 75.7 | 74.4 | 0 | 70.8 | 80.4 |
| Bulimia nervosa | 58.4 | 0.5 | 52.4 | 66.6 | 55.5 | 0.5 | 48.5 | 64.1 | 64 | 0.1 | 59.1 | 70.8 | 62.4 | 0.3 | 56.5 | 70.8 | 62.2 | 0.2 | 57.2 | 69.7 | 68 | 0.1 | 64.1 | 74.6 |
| Cannabis use disorders | 62.6 | 0.4 | 57.1 | 70.7 | 59.5 | 0.6 | 53.2 | 68.1 | 68.1 | 0.1 | 63.3 | 74.5 | 66.8 | 0.2 | 61.4 | 74.8 | 65.9 | 0.2 | 61.1 | 73.5 | 71.9 | 0.1 | 68.2 | 78.2 |
| Caries of deciduous teeth | 63 | 1.6 | 57.5 | 71.8 | 59.6 | 1.9 | 52.7 | 69.1 | 68.8 | 0.5 | 63.7 | 75.6 | 67.2 | 0.9 | 61.6 | 75.8 | 66.9 | 0.5 | 61.4 | 74.7 | 73.1 | 0.2 | 68.7 | 79.4 |
| Caries of permanent teeth | 64.3 | 0.3 | 58.3 | 71.8 | 61.4 | 0.4 | 54.3 | 69.1 | 69.9 | 0 | 64.4 | 75.6 | 68.5 | 0.2 | 62.4 | 76.4 | 68.2 | 0.1 | 63.6 | 74.6 | 74 | 0 | 70.2 | 79.4 |
| Cataract | 68.3 | 0 | 64.1 | 73.7 | 68 | 0 | 63.9 | 73.1 | 74 | 0 | 70.8 | 78.2 | 73.9 | 0 | 70.6 | 78.5 | 73.6 | 0 | 70.8 | 77.8 | 77.8 | 0 | 75.3 | 81.5 |
| Cellulitis | 64.2 | 0.9 | 58.3 | 72.4 | 61.1 | 1.3 | 54.3 | 69.7 | 70.3 | 0.2 | 65.2 | 76.3 | 68.9 | 0.4 | 63.1 | 76.4 | 69.8 | 0.1 | 65.8 | 75.9 | 74.6 | 0.1 | 70.9 | 80.6 |
| Cervical cancer | 60.3 | 0.1 | 51.8 | 69.4 | 57.7 | 0.1 | 49.1 | 66.6 | 65.8 | 0 | 59.3 | 74.5 | 64.8 | 0 | 56.4 | 74.1 | 65.8 | 0 | 59.6 | 73.7 | 71.8 | 0 | 67.6 | 79.1 |
| Chronic kidney disease due to diabetes mellitus type 1 | 47.5 | 3 | 39 | 57.2 | 36.4 | 4.4 | 30.4 | 41.8 | 49.6 | 0.9 | 41 | 59.1 | 37.1 | 3.3 | 30.3 | 42.4 | 62.9 | 0.1 | 58.3 | 69.2 | 69.2 | 0 | 66 | 74.2 |
| Chronic kidney disease due to diabetes mellitus type 2 | 67.5 | 0.1 | 63 | 73 | 65.5 | 0 | 60.7 | 71.1 | 72.2 | 0 | 68.2 | 76.8 | 70 | 0 | 65.3 | 75.7 | 71 | 0 | 67.6 | 75.5 | 75.8 | 0 | 72.7 | 79.9 |
| Chronic kidney disease due to glomerulonephritis | 64.6 | 1.5 | 59.9 | 72.5 | 62.5 | 1 | 56.9 | 70.4 | 71.8 | 0.1 | 67.9 | 77 | 67.2 | 0.3 | 60.9 | 75 | 71.6 | 0 | 68.4 | 76.3 | 76.6 | 0 | 73.8 | 81.1 |
| Chronic kidney disease due to hypertension | 57.4 | 0.9 | 51.6 | 65 | 54.3 | 1 | 48 | 62.1 | 63.1 | 0.2 | 58 | 69.8 | 58.2 | 0.4 | 52 | 65.7 | 62.2 | 0.1 | 56.8 | 69.3 | 70.1 | 0 | 66.5 | 75.5 |
| Chronic kidney disease due to other and unspecified causes | 66.3 | 0.6 | 61.9 | 73.1 | 64.4 | 0.8 | 59.8 | 71.2 | 72.4 | 0.1 | 68.3 | 77.5 | 71 | 0.2 | 66.8 | 77.1 | 71.8 | 0 | 68 | 76.3 | 76.7 | 0 | 73.8 | 81.2 |
| Chronic lymphoid leukemia | 63.8 | 0.3 | 58.7 | 70.5 | 62.2 | 0.4 | 57 | 69 | 68.6 | 0.2 | 63.5 | 75.1 | 68.8 | 0.2 | 63.8 | 75.6 | 70 | 0 | 65.8 | 75.4 | 75.1 | 0 | 71.9 | 80.2 |
| Chronic myeloid leukemia | 49.1 | 4.3 | 34.1 | 63.5 | 51 | 1.5 | 39.8 | 62.2 | 64.4 | 0.4 | 58.4 | 73.7 | 59.4 | 0.8 | 49.6 | 71.1 | 64 | 0.2 | 57.6 | 72.6 | 71.2 | 0.1 | 66.8 | 78.4 |
| Chronic obstructive pulmonary disease | 61.3 | 0.4 | 56 | 68.2 | 62.6 | 0.2 | 57.3 | 69.3 | 69.4 | 0 | 65.2 | 74.6 | 69.9 | 0.1 | 65.4 | 76 | 68.1 | 0 | 64.2 | 73.3 | 75 | 0 | 71.6 | 79.9 |
| Cirrhosis and other chronic liver diseases due to alcohol use | 57.5 | 0 | 51.6 | 64.9 | 56.3 | 0 | 49.3 | 63.4 | 67.5 | 0 | 62.5 | 74.4 | 63.5 | 0 | 56.6 | 71.6 | 61.7 | 0 | 54.3 | 69.4 | 70.9 | 0 | 66.4 | 77.6 |
| Cirrhosis and other chronic liver diseases due to hepatitis B | 64.8 | 0 | 59.1 | 71.8 | 60.7 | 0 | 53.5 | 68.3 | 69.5 | 0 | 64.4 | 75.6 | 68.2 | 0 | 62.4 | 75.8 | 68.9 | 0 | 64.3 | 75.2 | 73.9 | 0 | 70.2 | 79.9 |
| Cirrhosis and other chronic liver diseases due to hepatitis C | 62.3 | 0.1 | 55 | 70.3 | 59.6 | 0 | 51.8 | 67.5 | 69.1 | 0 | 63.6 | 75.5 | 63.5 | 0 | 55.7 | 72.3 | 66.5 | 0 | 60.5 | 73.7 | 72 | 0 | 67.4 | 78.7 |
| Cirrhosis and other chronic liver diseases due to other causes | 53.9 | 3.9 | 47.1 | 64.2 | 53.1 | 4.5 | 46.5 | 63.4 | 66 | 0.9 | 61 | 73.8 | 61.4 | 1.4 | 54.7 | 71.2 | 61 | 0.4 | 53.3 | 70.4 | 70.1 | 0.3 | 65.4 | 77 |
| Cocaine use disorders | 52.2 | 0.6 | 42.9 | 61.9 | 53.9 | 0.7 | 47.5 | 62.1 | 61.1 | 0.1 | 56.2 | 68.5 | 60.5 | 0.5 | 55.1 | 68.3 | 57.6 | 0.5 | 50.3 | 66.7 | 65.9 | 0.1 | 62.4 | 71.8 |
| Colon and rectum cancer | 60.4 | 0.3 | 53.4 | 69.5 | 59.7 | 0.2 | 53.3 | 67.9 | 69.2 | 0 | 64.4 | 75.3 | 68 | 0.1 | 62.8 | 75.4 | 69.5 | 0 | 65 | 75.6 | 74.8 | 0 | 71 | 80.3 |
| Conduct disorder | 61.8 | 1.2 | 56.1 | 70.5 | 58.5 | 1.4 | 51.9 | 67.8 | 67.3 | 0.4 | 62.5 | 74.3 | 65.7 | 0.9 | 60.1 | 74.6 | 65.5 | 0.5 | 60.7 | 73.4 | 71.6 | 0.2 | 67.8 | 78.2 |
| Conflict and terrorism | 65.4 | 0.1 | 59.1 | 72.4 | 62.4 | 0.1 | 55.9 | 69.8 | 70.4 | 0 | 65.2 | 76.3 | 69.8 | 0 | 63.9 | 76.5 | 68.8 | 0 | 64.4 | 75.3 | 74.3 | 0.1 | 71 | 80 |
| Congenital heart anomalies | 46.7 | 17.1 | 32.8 | 64.5 | 28.1 | 40.4 | 1.4 | 46.7 | 43.2 | 24.9 | 19.6 | 66.6 | 31.6 | 38.1 | 3.3 | 53.3 | 50.4 | 9.1 | 36.7 | 67 | 62.6 | 4.2 | 56 | 74.5 |
| Congenital musculoskeletal and limb anomalies | 50.5 | 8.2 | 46.3 | 60.8 | 46.8 | 10.6 | 41.4 | 58.4 | 57.9 | 1.9 | 54.2 | 64.5 | 56.1 | 3.5 | 51.1 | 64.7 | 56.4 | 2.1 | 52.4 | 63.9 | 62 | 1 | 58.7 | 67.9 |
| Contact dermatitis | 64 | 0.2 | 58.5 | 71.6 | 61.5 | 0.2 | 55.6 | 69.2 | 69.9 | 0 | 65.6 | 75.7 | 68.7 | 0.1 | 63.7 | 75.9 | 68.6 | 0.1 | 64.6 | 74.9 | 74.4 | 0 | 71.1 | 79.8 |
| Cyclist road injuries | 64.1 | 0.9 | 58.3 | 72.4 | 60.2 | 2.4 | 54.3 | 69.7 | 70 | 0.1 | 65.2 | 76.2 | 68.7 | 0.6 | 63.1 | 76.4 | 68.3 | 0.2 | 63.6 | 75.2 | 74.9 | 0.1 | 71.7 | 80.6 |
| Decubitus ulcer | 67.5 | 0.6 | 62.7 | 74.2 | 65.2 | 0.3 | 60.4 | 72.4 | 74.6 | 0 | 71.7 | 79 | 73.5 | 0.1 | 70.1 | 79.1 | 73.5 | 0 | 70.5 | 78.1 | 77.7 | 0 | 75.1 | 81.7 |
| Diabetes mellitus type 1 | 42.9 | 3.9 | 37.7 | 49.7 | 36 | 9.3 | 30.4 | 43.2 | 52 | 2.5 | 47.9 | 58.5 | 42.2 | 4.9 | 35.1 | 50.4 | 59.3 | 0.6 | 53.1 | 67.7 | 66.4 | 0.4 | 62.5 | 73.2 |
| Diabetes mellitus type 2 | 62.7 | 0.1 | 57.5 | 69.5 | 60.1 | 0.1 | 54.3 | 67.1 | 67.7 | 0 | 63.2 | 73.4 | 66 | 0 | 60.6 | 73 | 67.7 | 0 | 63.5 | 73.3 | 73.4 | 0 | 70.1 | 78.4 |
| Digestive congenital anomalies | 47.5 | 22.7 | 31.3 | 67.3 | 37.6 | 35.7 | 0.5 | 62.5 | 57.9 | 12.4 | 56.1 | 71.6 | 57.7 | 10.8 | 53.3 | 71.7 | 61.9 | 3.6 | 57.9 | 71.3 | 68.7 | 1.7 | 65.6 | 75.9 |
| Down syndrome | 36.3 | 36.1 | 1.3 | 60.5 | 35 | 32.9 | 2.2 | 55.2 | 48.3 | 18.3 | 36.4 | 66.5 | 50.9 | 14.2 | 41.8 | 67.7 | 52.8 | 5.5 | 45.2 | 65.3 | 65.4 | 2.6 | 62.1 | 73.5 |
| Drowning | 43.6 | 34.6 | 4.2 | 69.6 | 40 | 36.1 | 6.1 | 65.3 | 61.6 | 13.3 | 60.4 | 76.2 | 55.2 | 18.3 | 35.1 | 75.1 | 59.2 | 13.2 | 56 | 74.6 | 74.5 | 1.9 | 72.4 | 80.7 |
| Dysthymia | 64.9 | 0.2 | 59.1 | 72.4 | 62.3 | 0.2 | 55.9 | 69.7 | 70.5 | 0 | 65.9 | 76.2 | 69.5 | 0.1 | 63.9 | 76.4 | 68.9 | 0 | 64.4 | 75.2 | 74.7 | 0 | 70.9 | 80 |
| Edentulism and severe tooth loss | 66.4 | 0 | 61.6 | 72.9 | 65 | 0 | 60.3 | 70.8 | 72.1 | 0 | 68.1 | 76.8 | 70.8 | 0 | 66.2 | 76.8 | 69.9 | 0 | 65.8 | 75.2 | 75.4 | 0 | 72.1 | 80.1 |
| Endocarditis | 30.5 | 34.8 | 14.9 | 43.2 | 32.2 | 37.1 | 10.5 | 51.8 | 51.4 | 8.7 | 36.7 | 67.3 | 45.9 | 16.5 | 27.9 | 66.3 | 60.6 | 1.8 | 54 | 71.2 | 68.5 | 1.6 | 64.4 | 77.7 |
| Endometriosis | 62 | 0.1 | 56 | 70 | 60.2 | 0.1 | 53.5 | 68 | 67.8 | 0 | 62.8 | 74.3 | 67.1 | 0 | 61.3 | 74.8 | 65.9 | 0 | 60.8 | 73.1 | 71.8 | 0 | 67.9 | 78.1 |
| Environmental heat and cold exposure | 63.5 | 1.8 | 58.3 | 72.4 | 61.4 | 1.4 | 55.1 | 69.8 | 70.6 | 0.2 | 66 | 76.3 | 69.7 | 0.5 | 64.7 | 77.1 | 70 | 0.1 | 65.8 | 75.9 | 75.5 | 0 | 71.7 | 80.7 |
| Epilepsy | 36.9 | 14 | 26.3 | 46.4 | 39.7 | 8.2 | 30.6 | 48.6 | 56.8 | 1.8 | 50.6 | 65.7 | 53.6 | 2 | 46.8 | 61.8 | 58.9 | 0.7 | 53.4 | 66.7 | 64.4 | 0.6 | 59.2 | 72.5 |
| Esophageal cancer | 58.1 | 0.2 | 51.6 | 65.8 | 55.9 | 0.2 | 49.3 | 62.3 | 63.7 | 0 | 58.5 | 70 | 60.4 | 0.1 | 53.2 | 68.5 | 61.2 | 0 | 54.9 | 67.9 | 69.2 | 0 | 63.1 | 75.9 |
| Executions and police conflict | 63.7 | 0.6 | 57.5 | 71.7 | 60.3 | 1.4 | 53.5 | 69 | 69.5 | 0.1 | 64.4 | 75.6 | 67.7 | 0.4 | 61.6 | 75.8 | 67.5 | 0.2 | 62.1 | 74.6 | 73.7 | 0.4 | 70.2 | 80 |
| Exposure to forces of nature | 63.2 | 2 | 57.5 | 71.7 | 60.8 | 1 | 54.3 | 69 | 69.7 | 0.5 | 64.4 | 75.6 | 67.3 | 1.9 | 62.4 | 75.8 | 68.1 | 0.6 | 63.6 | 75.3 | 74.2 | 0.2 | 71 | 80 |
| Falls | 64.9 | 0.7 | 59.1 | 72.5 | 62.2 | 0.9 | 55.9 | 70.5 | 70.3 | 0.2 | 65.2 | 76.3 | 70.8 | 0.3 | 66.3 | 77.8 | 70.2 | 0.1 | 65.8 | 75.9 | 75.9 | 0 | 72.4 | 81.3 |
| Female infertility | 64.5 | 0 | 58.3 | 71.7 | 61.4 | 0 | 54.3 | 69 | 69.4 | 0 | 64.4 | 75.6 | 68.5 | 0 | 62.3 | 75.8 | 67.6 | 0 | 62.1 | 74.6 | 73.6 | 0 | 69.4 | 79.4 |
| Fire, heat, and hot substances | 63.4 | 1.6 | 57.5 | 71.7 | 59.1 | 3.5 | 53.4 | 69 | 69.4 | 0.3 | 64.4 | 75.6 | 68 | 0.9 | 62.4 | 76.4 | 68.1 | 0.3 | 62.9 | 74.6 | 73.8 | 0.1 | 70.2 | 79.4 |
| Foreign body in eyes | 64.1 | 0.7 | 58.3 | 72.4 | 62.5 | 0.8 | 56.7 | 70.5 | 70.2 | 0 | 65.2 | 76.2 | 70.3 | 0.1 | 65.5 | 77.1 | 68.4 | 0.1 | 63.6 | 75.2 | 74.2 | 0 | 70.9 | 80 |
| Foreign body in other body part | 63.3 | 1.6 | 57.5 | 71.7 | 60.2 | 3.1 | 54.3 | 69.7 | 69.9 | 0.3 | 65.2 | 76.3 | 68.9 | 0.8 | 63.9 | 76.4 | 69.9 | 0.2 | 65.8 | 75.9 | 75.6 | 0.1 | 72.4 | 80.7 |
| Fungal skin diseases | 64.3 | 1 | 58.3 | 72.5 | 62.2 | 0.8 | 55.9 | 70.5 | 71.7 | 0 | 67.6 | 77 | 70.4 | 0.2 | 65.5 | 77.2 | 71.1 | 0.1 | 67.2 | 76.5 | 76.5 | 0 | 73.2 | 81.3 |
| G6PD deficiency | 59.5 | 6.9 | 54.3 | 71.1 | 56.1 | 7.7 | 50.2 | 68.3 | 68.1 | 1.5 | 63.7 | 75.6 | 66 | 2.4 | 60 | 75.8 | 66.4 | 1.2 | 61.4 | 74.6 | 72.7 | 0.5 | 68.7 | 79.4 |
| G6PD trait | 59.6 | 6.9 | 55.1 | 71.1 | 56.1 | 7.7 | 50.2 | 68.3 | 68.1 | 1.5 | 63.7 | 75.6 | 66.3 | 2.2 | 60.8 | 75.8 | 66.5 | 1.1 | 61.4 | 74.7 | 72.9 | 0.5 | 68.7 | 79.4 |
| Gallbladder and biliary diseases | 66.1 | 0.2 | 60.7 | 73.2 | 63.2 | 0.1 | 56.8 | 70.5 | 71.7 | 0 | 67.6 | 77 | 70.4 | 0 | 64.8 | 77.2 | 70.9 | 0 | 67.2 | 75.9 | 76.2 | 0 | 73.2 | 80.7 |
| Gallbladder and biliary tract cancer | 56.7 | 0.4 | 50 | 64.4 | 56.3 | 0.2 | 49.7 | 63.4 | 62.9 | 0 | 57 | 70.3 | 60.4 | 0.1 | 53.3 | 67.7 | 67.3 | 0 | 61.9 | 73.9 | 71.9 | 0 | 67.1 | 77.7 |
| Gastritis and duodenitis | 64.7 | 0.4 | 59 | 72.3 | 62.5 | 0.3 | 56.6 | 69.6 | 71 | 0 | 66.6 | 76.1 | 70.2 | 0.1 | 65.3 | 77 | 70 | 0 | 66.3 | 75.7 | 74.6 | 0 | 70.8 | 79.9 |
| Gastroesophageal reflux disease | 65.4 | 0 | 59.1 | 72.4 | 62.7 | 0.1 | 56.7 | 69.8 | 70.7 | 0 | 66 | 76.3 | 69.8 | 0 | 63.9 | 76.5 | 69.6 | 0 | 65.1 | 75.3 | 75.2 | 0 | 71.7 | 80 |
| Genital prolapse | 65.9 | 0 | 60.6 | 72.4 | 62.8 | 0 | 57.4 | 69.7 | 71.1 | 0 | 66.7 | 76.3 | 70.6 | 0 | 65.5 | 77 | 70.5 | 0 | 66.5 | 75.8 | 75.4 | 0 | 71.7 | 80 |
| Glaucoma | 68.6 | 0 | 64.8 | 72.8 | 67.5 | 0 | 63.6 | 71.9 | 72.6 | 0 | 69.1 | 76.7 | 72.9 | 0 | 69.4 | 77.1 | 72.3 | 0 | 69.1 | 76.3 | 76.6 | 0 | 73.9 | 80.5 |
| Gout | 67.7 | 0 | 62.2 | 73.1 | 64.9 | 0 | 59.1 | 71.2 | 71.8 | 0 | 67.5 | 77 | 71.3 | 0 | 66.3 | 77.1 | 70.8 | 0 | 66.5 | 75.9 | 75.9 | 0 | 72.4 | 80.7 |
| Hodgkin lymphoma | 41.6 | 21.7 | 22 | 62.5 | 47.4 | 7.7 | 32.9 | 61.8 | 68.7 | 0.4 | 64.1 | 75.3 | 61.7 | 2.3 | 53.8 | 73.4 | 67.3 | 0.1 | 62.6 | 74.4 | 73.1 | 0.1 | 69.2 | 79.1 |
| Hypertensive heart disease | 56.1 | 3.1 | 49.2 | 66.7 | 57.3 | 1.5 | 50.1 | 66.6 | 68.3 | 0.3 | 64.1 | 73.9 | 68.2 | 0.9 | 63.7 | 75.2 | 66 | 0.3 | 61.2 | 73.3 | 74.6 | 0.1 | 71.5 | 79.2 |
| Idiopathic developmental intellectual disability | 61.7 | 1.1 | 56.4 | 69.9 | 59 | 1 | 52.8 | 67.4 | 68.1 | 0.1 | 63.5 | 74.2 | 66.5 | 0.4 | 61.3 | 74.3 | 66.4 | 0.2 | 62.1 | 73.1 | 72.2 | 0 | 68.7 | 78.1 |
| Inflammatory bowel disease | 46.3 | 3.4 | 39.3 | 54.9 | 35.5 | 11.9 | 25.2 | 45 | 65.2 | 0.2 | 61.3 | 70.7 | 59 | 1.6 | 52.2 | 68.7 | 62.5 | 0.1 | 58.2 | 68.7 | 66.6 | 0.1 | 62.9 | 72 |
| Inguinal, femoral, and abdominal hernia | 61.7 | 0.8 | 56.9 | 69.2 | 60.4 | 0.7 | 55.2 | 67.6 | 68.7 | 0.1 | 64.6 | 74.2 | 67.8 | 0.2 | 63.4 | 74 | 69.1 | 0 | 65.4 | 74.4 | 73.3 | 0 | 70.1 | 77.9 |
| Interstitial lung disease and pulmonary sarcoidosis | 64.3 | 1.7 | 60.3 | 71.5 | 58.3 | 3.2 | 52.2 | 68.1 | 69.7 | 0.2 | 65.8 | 75 | 65.8 | 0.9 | 59.9 | 74.1 | 66.5 | 0.2 | 62.2 | 72.4 | 72.2 | 0.1 | 68.6 | 77.1 |
| Intracerebral hemorrhage | 55.6 | 1.6 | 48.3 | 64.7 | 54.4 | 0.6 | 47.4 | 61.5 | 61.8 | 0.1 | 56.2 | 68.2 | 61.7 | 0.4 | 55.6 | 68.9 | 65.4 | 0.1 | 60.6 | 71.9 | 67.7 | 0 | 63.1 | 73.9 |
| Ischemic heart disease | 69 | 0.1 | 64.5 | 74.4 | 67.1 | 0 | 62.3 | 72.6 | 73.2 | 0 | 69.1 | 77.7 | 73.1 | 0 | 69.3 | 78.4 | 72 | 0 | 68.6 | 76.5 | 77 | 0 | 73.9 | 81.3 |
| Ischemic stroke | 62.9 | 0.5 | 58 | 69.6 | 62.2 | 0.5 | 57.5 | 68.7 | 68.5 | 0 | 64.4 | 73.3 | 69.4 | 0.1 | 65.4 | 74.8 | 70.1 | 0 | 66.6 | 74.9 | 73.5 | 0 | 70.1 | 77.9 |
| Kidney cancer | 61.8 | 4.3 | 57.3 | 70.9 | 60.5 | 2.3 | 54.9 | 68.9 | 68.1 | 0.4 | 62.7 | 74.8 | 67.4 | 0.6 | 61.5 | 74.9 | 69.1 | 0.1 | 64.9 | 75.1 | 72.3 | 0 | 67.8 | 78.4 |
| Klinefelter syndrome | 59.3 | 6.9 | 54.8 | 70.7 | 55.8 | 7.7 | 49.9 | 68 | 67.8 | 1.5 | 63.3 | 75.2 | 65.9 | 2.2 | 60.4 | 75.4 | 66.1 | 1.1 | 61.1 | 74.3 | 72.5 | 0.5 | 68.3 | 79 |
| Larynx cancer | 59.4 | 0.1 | 51.8 | 67.5 | 59.3 | 0.1 | 53.1 | 66.4 | 68.3 | 0 | 62.8 | 74.5 | 67.1 | 0 | 61.2 | 74.1 | 68.8 | 0 | 64.1 | 74.9 | 74.9 | 0 | 71.4 | 79.7 |
| Lip and oral cavity cancer | 60.2 | 1.3 | 54.1 | 69.6 | 59.6 | 0.2 | 52.7 | 67.6 | 67.4 | 0 | 61.7 | 74.5 | 66.4 | 0.1 | 59.6 | 74.9 | 69.4 | 0 | 64.8 | 75.3 | 74.2 | 0 | 70.7 | 79.9 |
| Liver cancer due to alcohol use | 55.6 | 0.1 | 48.2 | 63.5 | 56.3 | 0 | 50 | 63.2 | 59.9 | 0 | 52.8 | 66.7 | 60.6 | 0 | 54.5 | 67.7 | 60.8 | 0 | 54.4 | 67.2 | 68.8 | 0 | 63.8 | 74.6 |
| Liver cancer due to hepatitis B | 46.9 | 6.9 | 36.3 | 58.9 | 50.9 | 1.9 | 43.5 | 59.2 | 56.7 | 0.2 | 48.4 | 64.7 | 54.8 | 1.8 | 47.4 | 64.1 | 58.2 | 0.9 | 52 | 65.3 | 66.6 | 0.3 | 60.7 | 73.9 |
| Liver cancer due to hepatitis C | 58 | 0.3 | 52.3 | 65 | 58.2 | 0.1 | 52.5 | 65.1 | 61.9 | 0 | 56.1 | 69 | 62.6 | 0 | 56.5 | 69.2 | 61.6 | 0 | 55.1 | 68.3 | 70.1 | 0 | 65.1 | 75.8 |
| Liver cancer due to NASH | 55.9 | 1.7 | 49.9 | 64.5 | 57.5 | 0.4 | 51.4 | 64.9 | 61.6 | 0 | 55.4 | 69.6 | 62.5 | 0.3 | 56.5 | 70 | 62.3 | 0.1 | 56 | 68.9 | 70.7 | 0 | 66.2 | 76.4 |
| Liver cancer due to other causes | 45.7 | 13.4 | 33.5 | 59.8 | 51.9 | 3 | 45 | 60.9 | 58 | 0.3 | 49.8 | 66.7 | 57 | 2.3 | 49.9 | 66.4 | 58.9 | 1.1 | 52.7 | 66.5 | 68.8 | 0.3 | 63.5 | 75.7 |
| Low back pain | 63.1 | 0.1 | 57.9 | 69.8 | 59.9 | 0.2 | 54.2 | 67.1 | 68.5 | 0 | 64.2 | 74 | 66.9 | 0.1 | 62 | 73.6 | 67.7 | 0 | 63.8 | 73.5 | 72.7 | 0 | 69.3 | 77.9 |
| Major depressive disorder | 65.1 | 0.2 | 59.7 | 72.3 | 62.2 | 0.2 | 55.8 | 69.6 | 70.9 | 0 | 66.6 | 76.2 | 69.5 | 0.1 | 63.8 | 76.3 | 68.4 | 0.1 | 63.5 | 75.1 | 74.6 | 0 | 70.8 | 79.9 |
| Male infertility | 64.4 | 0 | 58.3 | 72.3 | 61.2 | 0 | 54.3 | 69.6 | 69.4 | 0 | 64.3 | 75.6 | 68.4 | 0 | 63 | 75.8 | 67.6 | 0 | 62.8 | 74.6 | 73.5 | 0 | 69.4 | 79.9 |
| Malignant skin melanoma | 56.2 | 1 | 45.5 | 67.9 | 56.6 | 0.8 | 47.7 | 66.8 | 67.9 | 0.1 | 62.6 | 75.3 | 66.9 | 0.1 | 60.5 | 75.5 | 70 | 0 | 65.8 | 75.7 | 75 | 0 | 71.5 | 80.6 |
| Mesothelioma | 55 | 1.5 | 46.7 | 65.2 | 51.3 | 1.4 | 42.6 | 61.5 | 57.9 | 0.3 | 49.2 | 67.9 | 58.8 | 0.5 | 51.1 | 67.6 | 64.2 | 0 | 59.1 | 70.5 | 67.4 | 0 | 62 | 73.9 |
| Migraine | 62.8 | 0.4 | 57.3 | 70.7 | 59.8 | 0.6 | 53.5 | 68.2 | 68.6 | 0.1 | 63.9 | 74.7 | 67 | 0.3 | 61.7 | 74.9 | 66.7 | 0.1 | 62.1 | 73.8 | 72.5 | 0 | 69 | 78.5 |
| Motor neuron disease | 54.9 | 2.5 | 46.3 | 65.7 | 38.2 | 22.3 | 23.8 | 55.5 | 59.5 | 2.9 | 53.5 | 69.3 | 49 | 7.2 | 40 | 60.7 | 58.7 | 1.7 | 54 | 65.9 | 63.9 | 0.5 | 59.3 | 70.3 |
| Motor vehicle road injuries | 62.1 | 4.1 | 57.5 | 71.7 | 52.7 | 12.4 | 40.9 | 68.3 | 69.3 | 0.4 | 64.4 | 75.6 | 68 | 1.1 | 62.3 | 76.4 | 68.1 | 0.5 | 63.6 | 75.2 | 74.4 | 0.2 | 70.9 | 80 |
| Motorcyclist road injuries | 63.2 | 1.9 | 57.5 | 71.7 | 59.4 | 2.8 | 53.4 | 69 | 69.7 | 0.2 | 64.4 | 76.2 | 67.7 | 1.1 | 62.3 | 76.4 | 68.2 | 0.1 | 63.6 | 74.6 | 74.2 | 0.1 | 70.9 | 80 |
| Multiple myeloma | 57.1 | 0.5 | 50.8 | 64.9 | 55.2 | 0.4 | 49.1 | 62.2 | 64.1 | 0.2 | 58.8 | 71.1 | 61 | 0.2 | 54.4 | 68 | 67 | 0 | 62.2 | 72.7 | 71.6 | 0 | 67.4 | 77.2 |
| Multiple sclerosis | 48.4 | 0.5 | 42.2 | 55.5 | 45.1 | 0.6 | 38.2 | 51.9 | 53.4 | 0.3 | 44.5 | 62.7 | 49.3 | 0.4 | 40.8 | 57.7 | 52.7 | 0.1 | 46.7 | 59.5 | 58.1 | 0.1 | 51.9 | 65.3 |
| Myelodysplastic, myeloproliferative, and other hematopoietic neoplasms | 49.7 | 18.5 | 38.8 | 67.5 | 47.6 | 14.8 | 37.6 | 63 | 68.2 | 1.8 | 64.2 | 75.5 | 58.8 | 6.8 | 51.5 | 72.1 | 70.6 | 0.3 | 66.5 | 75.8 | 73.7 | 0.5 | 70.1 | 79.4 |
| Myocarditis | 57.5 | 1.6 | 48.2 | 68.6 | 48.2 | 3.3 | 36.9 | 60.5 | 63.7 | 1 | 59.4 | 71.6 | 65 | 0.9 | 59.2 | 74.3 | 63 | 0.8 | 56.8 | 72.8 | 72 | 0.3 | 68.8 | 78 |
| Nasopharynx cancer | 56.5 | 3.4 | 47.4 | 67.8 | 55.4 | 2.3 | 47.4 | 65.3 | 63.2 | 0.2 | 55.1 | 72.4 | 62.2 | 1.9 | 53.8 | 72.6 | 63.6 | 0.3 | 55.7 | 72.3 | 68 | 0.2 | 61.5 | 76.3 |
| Near vision loss | 66.2 | 0.1 | 61.2 | 73 | 64.1 | 0 | 58.3 | 71 | 71.8 | 0 | 67.5 | 76.9 | 70.9 | 0 | 66.2 | 77.2 | 71 | 0 | 67.2 | 76.3 | 76 | 0 | 73 | 81 |
| Neck pain | 63.3 | 0.1 | 58.2 | 69.9 | 61.2 | 0 | 55.7 | 68 | 68.6 | 0 | 64.3 | 74 | 67.8 | 0 | 63.1 | 74.2 | 67.8 | 0 | 63.8 | 73.5 | 73.2 | 0 | 70 | 78.2 |
| Neural tube defects | 19.7 | 57.9 | 0.4 | 44.6 | 5.7 | 88 | 0 | 0.4 | 37.5 | 23.2 | 27.7 | 52 | 28.5 | 41.5 | 0.4 | 50.3 | 19.8 | 58.1 | 0.4 | 44.8 | 44.5 | 14.1 | 43.4 | 55.6 |
| Non-Hodgkin lymphoma | 48.9 | 14.2 | 34.3 | 65.6 | 50.2 | 9 | 39.8 | 63.7 | 65.7 | 0.9 | 60.1 | 73.8 | 61.6 | 2.7 | 54.7 | 72.6 | 69.4 | 0.1 | 65.1 | 75.2 | 73.8 | 0.1 | 70.1 | 79.1 |
| Non-melanoma skin cancer (basal-cell carcinoma) | 66.4 | 0 | 60.7 | 73.1 | 65.4 | 0 | 59.9 | 71.9 | 72.7 | 0 | 68.3 | 77.7 | 72.1 | 0 | 67.8 | 77.8 | 72 | 0 | 68.6 | 76.5 | 77 | 0 | 73.9 | 81.3 |
| Non-melanoma skin cancer (squamous-cell carcinoma) | 55 | 3.3 | 45.9 | 66 | 56.7 | 1.6 | 48.6 | 66.1 | 63.3 | 0.3 | 56.9 | 71.8 | 73.4 | 0.1 | 70 | 78.9 | 72.6 | 0 | 69.2 | 77 | 75.1 | 0 | 72.4 | 80.7 |
| Non-rheumatic calcific aortic valve disease | 68.6 | 0 | 64.5 | 74.2 | 65.3 | 0.1 | 60.6 | 71.2 | 72.4 | 0 | 68.2 | 77.4 | 71.7 | 0 | 67.7 | 77.5 | 72.7 | 0 | 69.3 | 76.9 | 77 | 0 | 74.5 | 81 |
| Non-rheumatic degenerative mitral valve disease | 68 | 0 | 63.7 | 73.7 | 64.7 | 0 | 59.8 | 71.2 | 72.5 | 0 | 68.9 | 77.4 | 72.2 | 0 | 68.4 | 77.7 | 71.9 | 0 | 68.5 | 76.4 | 76.3 | 0 | 73.2 | 80.9 |
| Non-venomous animal contact | 64.3 | 1 | 58.3 | 72.5 | 61.1 | 1.3 | 54.3 | 69.8 | 70 | 0.2 | 65.2 | 76.3 | 69.3 | 0.4 | 64 | 76.5 | 68.8 | 0.1 | 64.4 | 75.3 | 74.5 | 0 | 71 | 80 |
| Opioid use disorders | 48.6 | 0.3 | 43.8 | 53.9 | 47 | 0.4 | 41.4 | 52.8 | 53.5 | 0.1 | 47.9 | 58.8 | 51.6 | 0.3 | 45.6 | 57.8 | 43.7 | 1.5 | 34 | 52.2 | 58.7 | 0.1 | 52.5 | 65.2 |
| Orofacial clefts | 56.1 | 10.1 | 51.9 | 69.1 | 53.8 | 10 | 47.7 | 67.2 | 66.1 | 1.9 | 62 | 73.7 | 64.3 | 2.8 | 59.2 | 73.9 | 65 | 1.3 | 60.1 | 73.1 | 71.3 | 0.5 | 67.2 | 77.7 |
| Osteoarthritis | 66.6 | 0 | 61.7 | 72.7 | 64.3 | 0 | 59 | 70.4 | 70.2 | 0 | 65.7 | 75.7 | 70.4 | 0 | 65.8 | 76.5 | 69.8 | 0 | 65.6 | 75.1 | 74.7 | 0 | 71.2 | 79.8 |
| Other benign and in situ neoplasms | 65.3 | 0.9 | 59.9 | 72.5 | 62.8 | 1 | 57.6 | 70.5 | 70.8 | 0.1 | 66 | 76.3 | 70.1 | 0.4 | 64.8 | 77.2 | 70.2 | 0 | 65.8 | 75.3 | 74.9 | 0.1 | 71 | 80.1 |
| Other cardiomyopathy | 50.1 | 6.4 | 34.9 | 66.1 | 40.8 | 12.7 | 26.2 | 55.9 | 61.1 | 2.3 | 56.3 | 70.1 | 57.8 | 3.8 | 48.2 | 70.8 | 60 | 2.7 | 53.2 | 71.2 | 70.8 | 0.8 | 67.5 | 77.7 |
| Other cardiovascular and circulatory diseases | 63.2 | 0.5 | 57.4 | 71.6 | 62 | 0.3 | 56.6 | 69.7 | 70.8 | 0 | 67.1 | 75.6 | 70.5 | 0 | 66.4 | 76.3 | 70.7 | 0 | 67.5 | 75.4 | 75.7 | 0 | 72.9 | 80 |
| Other chromosomal abnormalities | 52.8 | 9.7 | 48.5 | 64.9 | 37.9 | 31.2 | 0.5 | 58.9 | 60.1 | 4.5 | 55.8 | 69.2 | 53.3 | 12.7 | 46.9 | 68.9 | 25.3 | 57.8 | 0 | 58.6 | 63.1 | 6.6 | 60.6 | 73.6 |
| Other congenital birth defects | 41.8 | 22 | 30.4 | 58.4 | 16.3 | 68.9 | 0 | 40 | 55.9 | 9.4 | 53.6 | 66.8 | 34.6 | 40.8 | 0.4 | 61.4 | 43.9 | 21.6 | 30.4 | 60.9 | 50.9 | 14 | 49.1 | 63.9 |
| Other exposure to mechanical forces | 63.6 | 1.5 | 57.5 | 71.7 | 61.1 | 1 | 54.3 | 69.8 | 69.8 | 0.2 | 64.4 | 75.6 | 68.8 | 0.3 | 63.2 | 76.5 | 68.5 | 0.1 | 63.6 | 75.3 | 74.3 | 0 | 71 | 80 |
| Other gynecological diseases | 63.9 | 0.1 | 57.9 | 71.8 | 61 | 0.1 | 54.2 | 69 | 69.2 | 0 | 64.2 | 75.5 | 68.4 | 0 | 62.8 | 76.1 | 67.5 | 0 | 62.5 | 74.6 | 73.2 | 0 | 69.3 | 79.3 |
| Other hemoglobinopathies and hemolytic anemias | 81 | 0 | 80.2 | 81.6 | 80.7 | 0 | 79.8 | 81.6 | 83.3 | 0 | 82.8 | 84 | 82.6 | 0 | 81.6 | 83.3 | 79.7 | 0 | 78.9 | 80.3 | 82.9 | 0 | 81.9 | 83.1 |
| Other leukemia | 42.6 | 32.1 | 12.6 | 66.3 | 50.2 | 14 | 39.1 | 65.7 | 62.9 | 4.8 | 57.9 | 74.4 | 56.5 | 10.4 | 44.4 | 72.7 | 66.9 | 1 | 62.7 | 74.3 | 71.7 | 0.5 | 67.6 | 78.3 |
| Other malignant neoplasms | 46.6 | 18.9 | 27.2 | 65.2 | 48.9 | 12.7 | 37.5 | 63.8 | 70.3 | 0.2 | 65.7 | 76.1 | 56.3 | 7.9 | 46 | 71.1 | 66.3 | 0.5 | 61 | 74.2 | 75.5 | 0.1 | 72.4 | 80.6 |
| Other oral disorders | 63.8 | 1 | 57.5 | 71.8 | 61 | 0.9 | 54.3 | 69.1 | 69.7 | 0.1 | 64.4 | 75.6 | 68.3 | 0.4 | 62.4 | 76.4 | 68.1 | 0.2 | 62.9 | 74.6 | 73.8 | 0 | 70.2 | 79.4 |
| Other pharynx cancer | 54 | 1 | 46.6 | 62.4 | 55.6 | 0.3 | 49.1 | 62.9 | 63.3 | 0.1 | 56.6 | 71.1 | 60.7 | 0.3 | 53.6 | 68.4 | 67 | 0 | 61.4 | 73.6 | 68.8 | 0 | 62.9 | 75.6 |
| Other pneumoconiosis | 60 | 0.5 | 54.3 | 67.3 | 60.2 | 0.9 | 54.4 | 69.5 | 67.1 | 0.1 | 62.7 | 73.4 | 68.8 | 0.1 | 64.9 | 74.8 | 66.7 | 0.1 | 62.6 | 72.7 | 72.1 | 0 | 68.6 | 76.6 |
| Other road injuries | 64.3 | 0.6 | 58.3 | 72.4 | 61.4 | 1.4 | 55.1 | 69.7 | 69.9 | 0.1 | 65.2 | 75.6 | 69.2 | 0.4 | 63.9 | 76.4 | 68.7 | 0.3 | 64.4 | 75.3 | 74.9 | 0 | 71.7 | 80 |
| Other skin and subcutaneous diseases | 65.1 | 0.7 | 59.9 | 72.5 | 63 | 0.6 | 56.8 | 70.5 | 71.3 | 0 | 66.8 | 76.3 | 70.3 | 0.2 | 65.5 | 77.2 | 70.4 | 0 | 65.8 | 75.9 | 75.8 | 0 | 72.5 | 80.7 |
| Other transport injuries | 63.9 | 1.3 | 58.3 | 72.4 | 56.8 | 7.8 | 51.8 | 69 | 70.4 | 0.2 | 65.2 | 76.3 | 68.7 | 1.3 | 63.9 | 77.1 | 69.5 | 0.2 | 65.1 | 75.9 | 75.7 | 0 | 72.4 | 80.7 |
| Other unintentional injuries | 63.9 | 1.3 | 58.3 | 72.4 | 61.1 | 1.7 | 55.1 | 69.8 | 70.3 | 0.2 | 65.2 | 76.3 | 69.2 | 0.4 | 63.9 | 77.1 | 69.4 | 0.1 | 65.1 | 75.3 | 74.8 | 0 | 71 | 80 |
| Other vision loss | 63.3 | 0.4 | 58.4 | 70.1 | 62 | 0.2 | 56.7 | 68.9 | 71 | 0 | 67.1 | 76.1 | 69.1 | 0.1 | 64.7 | 75.3 | 70.1 | 0 | 66.5 | 75.6 | 75.8 | 0 | 72.9 | 80.4 |
| Ovarian cancer | 58.7 | 0.5 | 50.7 | 67.7 | 57.2 | 0.3 | 49.3 | 66 | 64.1 | 0 | 57.1 | 72.2 | 63.3 | 0.2 | 54.7 | 73.2 | 65.2 | 0 | 59 | 72.6 | 70.3 | 0 | 64.3 | 78.2 |
| Pancreatic cancer | 57.8 | 0.3 | 51.7 | 65.2 | 56 | 0.2 | 49.7 | 62.9 | 61.1 | 0 | 55.3 | 68 | 59.9 | 0.1 | 52.4 | 67.5 | 62.6 | 0 | 56.6 | 69.3 | 68.5 | 0 | 63 | 75.2 |
| Pancreatitis | 65 | 0.4 | 59.1 | 72.4 | 63.4 | 0.2 | 57.5 | 70.5 | 71.2 | 0 | 66.7 | 76.9 | 70.1 | 0.1 | 64.7 | 77.1 | 70.1 | 0 | 65.8 | 75.9 | 75.1 | 0 | 71.7 | 80.6 |
| Paralytic ileus and intestinal obstruction | 41.7 | 23.2 | 21.9 | 61.2 | 39.1 | 29.5 | 9.8 | 61.2 | 64.2 | 4.5 | 60.3 | 74.1 | 52.5 | 15.1 | 41.2 | 70.7 | 68.6 | 0.5 | 64.8 | 75 | 70.6 | 0.4 | 65.4 | 77.3 |
| Parkinson's disease | 67.2 | 0 | 63.4 | 72.1 | 65.2 | 0 | 61.2 | 70.1 | 69.7 | 0 | 66 | 74.2 | 70.1 | 0 | 66.6 | 74.7 | 69.2 | 0 | 65.8 | 73.3 | 73.3 | 0 | 70.1 | 77.4 |
| Pedestrian road injuries | 63.6 | 2.1 | 58.3 | 72.4 | 56.1 | 10.1 | 50.2 | 69 | 69.7 | 0.7 | 65.2 | 76.2 | 67.6 | 2.3 | 63.1 | 76.4 | 68.4 | 0.5 | 64.3 | 75.2 | 74.9 | 0.2 | 71.7 | 80.6 |
| Peptic ulcer disease | 65.1 | 0.3 | 59.8 | 73 | 63.3 | 0.3 | 57.4 | 71.1 | 71.1 | 0 | 66.7 | 76.2 | 72.1 | 0.1 | 67.8 | 78.3 | 71 | 0 | 67.2 | 75.8 | 75.7 | 0 | 72.3 | 80.7 |
| Periodontal diseases | 66 | 0 | 60.7 | 72.5 | 63 | 0 | 56.8 | 69.8 | 71 | 0 | 66 | 76.3 | 69.8 | 0 | 64 | 76.5 | 70.2 | 0 | 65.8 | 75.3 | 75.4 | 0 | 71.7 | 80 |
| Peripheral artery disease | 68.8 | 0 | 63.8 | 73.8 | 66.8 | 0 | 61.5 | 71.9 | 72.3 | 0 | 68.2 | 77 | 72.4 | 0 | 67.8 | 77.7 | 71.6 | 0 | 67.9 | 76.4 | 76.4 | 0 | 73.2 | 80.7 |
| Physical violence by firearm | 59.4 | 6.1 | 54.2 | 71.7 | 45.8 | 15.9 | 23.5 | 65.3 | 69.8 | 0.3 | 65.2 | 76.3 | 55.4 | 9.1 | 31.5 | 74.4 | 64.5 | 3.1 | 60.7 | 74.6 | 74.1 | 0.1 | 70.2 | 80 |
| Physical violence by other means | 63.6 | 1.5 | 57.5 | 71.7 | 57.9 | 5.8 | 52.6 | 69 | 69.7 | 0.2 | 64.4 | 75.6 | 64.4 | 3.9 | 59.1 | 75.8 | 67.7 | 0.4 | 62.9 | 74.6 | 73.8 | 0.2 | 70.2 | 79.4 |
| Physical violence by sharp object | 63.9 | 1 | 58.3 | 71.8 | 58.9 | 2.8 | 52.7 | 69 | 69.4 | 0.2 | 64.4 | 75.6 | 67.5 | 1.1 | 62.4 | 76.4 | 67.6 | 0.2 | 62.2 | 74.6 | 73.8 | 0.1 | 70.2 | 80 |
| Poisoning by carbon monoxide | 61.8 | 3.7 | 56.6 | 71.7 | 51.3 | 15.8 | 42.6 | 68.2 | 66.5 | 3 | 62.8 | 75.6 | 63.5 | 7.4 | 59.1 | 75.7 | 67.1 | 1.2 | 62.8 | 75.2 | 73.9 | 0.2 | 70.2 | 80 |
| Poisoning by other means | 60 | 6.5 | 55 | 71.6 | 53.8 | 11.8 | 47.6 | 68.2 | 68.2 | 1.6 | 64.3 | 75.5 | 65.4 | 3.8 | 60.7 | 75.7 | 68.3 | 0.6 | 64.3 | 75.2 | 74.2 | 0.1 | 70.9 | 80 |
| Polycystic ovarian syndrome | 63.6 | 0.4 | 58 | 72.1 | 60.3 | 0.7 | 54 | 69 | 69.2 | 0.1 | 64.3 | 75.6 | 67.6 | 0.3 | 62.1 | 75.7 | 67.5 | 0.2 | 62.6 | 74.6 | 73.1 | 0.1 | 69.2 | 79.7 |
| Premenstrual syndrome | 64.2 | 0.2 | 58.3 | 71.8 | 61.2 | 0.2 | 54.3 | 69.1 | 69.5 | 0 | 64.4 | 75.6 | 68.3 | 0.1 | 62.4 | 75.8 | 67.7 | 0.1 | 62.2 | 74.6 | 73.5 | 0 | 69.5 | 79.4 |
| Prostate cancer | 66.7 | 0 | 62.7 | 71.4 | 66.2 | 0 | 61.9 | 71.1 | 72.1 | 0 | 68.3 | 76.6 | 72.3 | 0 | 68.3 | 77 | 71.3 | 0 | 67.2 | 75.6 | 76.3 | 0 | 72.9 | 80.7 |
| Pruritus | 64.7 | 0.7 | 59.1 | 72.5 | 62.3 | 0.6 | 55.9 | 69.8 | 70.9 | 0.1 | 66 | 76.3 | 69.7 | 0.2 | 64.7 | 76.5 | 69.7 | 0.1 | 65.1 | 75.3 | 75.4 | 0 | 71.7 | 80.7 |
| Psoriasis | 61.3 | 0.5 | 56.1 | 68.6 | 59.3 | 0.4 | 53.6 | 66.8 | 68 | 0 | 63.7 | 73.7 | 66.3 | 0.2 | 61.4 | 73.3 | 66.1 | 0.1 | 61.9 | 72.3 | 72 | 0 | 68.5 | 77.3 |
| Pulmonary aspiration and foreign body in airway | 53.6 | 16.6 | 47.5 | 70.3 | 32.6 | 48.2 | 0.5 | 63.8 | 67.6 | 3.1 | 63.6 | 76.2 | 40 | 39.8 | 3.4 | 70.8 | 67.4 | 4.5 | 65.1 | 75.9 | 75.1 | 1.5 | 73.1 | 81.3 |
| Pyoderma | 63.2 | 1.5 | 57.5 | 71.8 | 61.7 | 1.6 | 55.9 | 69.8 | 71 | 0.2 | 66.8 | 76.3 | 69.5 | 0.5 | 64 | 77.2 | 68.7 | 0.2 | 64.4 | 75.3 | 74.8 | 0.1 | 71 | 80 |
| Refraction disorders | 62.4 | 0.7 | 57.1 | 70.1 | 61.1 | 0.5 | 55.5 | 69 | 69.7 | 0.1 | 65.6 | 75.3 | 68.4 | 0.2 | 63.7 | 75.5 | 67.7 | 0.1 | 63.6 | 74.2 | 74.5 | 0 | 71.3 | 79.7 |
| Rheumatic heart disease | 58.2 | 1.8 | 53.6 | 66.2 | 48 | 4.3 | 40.8 | 57.7 | 61.2 | 0.6 | 57.4 | 67.4 | 61.1 | 1 | 55.3 | 69.6 | 66.2 | 0.3 | 60.8 | 74 | 73.4 | 0.1 | 70.2 | 78.7 |
| Rheumatoid arthritis | 61.5 | 0.3 | 56.4 | 68.2 | 59.1 | 0.3 | 53.7 | 66 | 67 | 0 | 62.8 | 72.3 | 65.5 | 0.1 | 60.6 | 72.1 | 66.4 | 0 | 62.5 | 71.8 | 71 | 0 | 67.3 | 76 |
| Scabies | 63.7 | 0.9 | 57.5 | 71.8 | 60.7 | 1 | 54.3 | 69.1 | 70 | 0.1 | 65.2 | 76.3 | 68.3 | 0.4 | 62.4 | 76.5 | 68.2 | 0.2 | 63.6 | 75.3 | 74.3 | 0.1 | 71 | 80 |
| Schizophrenia | 41.2 | 0.5 | 35.7 | 46 | 40.8 | 0.5 | 35.3 | 45.6 | 45.7 | 0.1 | 39.3 | 51.5 | 44.5 | 0.2 | 38.7 | 49.8 | 43.6 | 0.2 | 38.3 | 48 | 48.7 | 0 | 42.3 | 54.5 |
| Seborrhoeic dermatitis | 63.6 | 0.8 | 58.2 | 71.7 | 61.1 | 0.7 | 54.9 | 69.3 | 69.7 | 0.1 | 65.2 | 75.8 | 68.5 | 0.3 | 63.4 | 76.1 | 68.4 | 0.1 | 64.3 | 75 | 74.6 | 0 | 71.2 | 80 |
| Self-harm by firearm | 62.8 | 4.4 | 58.3 | 73.8 | 55.9 | 6.7 | 45.2 | 69 | 71.3 | 0.8 | 67.5 | 77 | 59.9 | 8.2 | 47.4 | 75.1 | 46.9 | 11.8 | 30 | 62.9 | 75.1 | 1 | 73.2 | 81.3 |
| Self-harm by other specified means | 60.2 | 5.8 | 54.2 | 71.7 | 53.1 | 9 | 38.4 | 67.6 | 67.2 | 2.5 | 62.8 | 75.6 | 53.4 | 14.3 | 27.9 | 74.3 | 66.4 | 1.3 | 61.4 | 74.6 | 70.6 | 1.5 | 67.9 | 79.4 |
| Sexual violence | 64.4 | 0.2 | 58.3 | 71.8 | 61.5 | 0.3 | 54.3 | 69.8 | 70 | 0 | 65.2 | 75.6 | 69 | 0.1 | 63.2 | 76.5 | 68.1 | 0.1 | 63.6 | 74.6 | 74.1 | 0 | 70.2 | 80 |
| Sickle cell disorders | 4 | 99.9 | 0.5 | 5.6 | 24.5 | 31.1 | 15.2 | 34 | 13.4 | 79.2 | 8.3 | 18.5 | 3.3 | 99.2 | 0.5 | 4 | 19.8 | 42.9 | 4.1 | 29 | 26.6 | 43.1 | 9.5 | 39.3 |
| Sickle cell trait | 59.5 | 6.9 | 55 | 70.9 | 56 | 7.7 | 50.1 | 68.2 | 68 | 1.5 | 63.5 | 75.5 | 66.2 | 2.2 | 60.7 | 75.6 | 66.4 | 1.1 | 61.4 | 74.6 | 72.8 | 0.5 | 68.6 | 79.3 |
| Silicosis | 57.9 | 0.2 | 52 | 64.5 | 55.3 | 0.9 | 48.1 | 64 | 65.6 | 0 | 60.2 | 72.7 | 64.4 | 0.1 | 59.1 | 71.1 | 65.3 | 0.1 | 61 | 71.2 | 71.2 | 0 | 67.6 | 75.3 |
| Stomach cancer | 54.7 | 0.5 | 45.8 | 64.4 | 55.7 | 0.2 | 48.5 | 64.3 | 65.3 | 0 | 59.6 | 72 | 62.9 | 0.1 | 55 | 72.2 | 66.5 | 0 | 61.2 | 73.7 | 72.4 | 0 | 67.8 | 78.5 |
| Subarachnoid hemorrhage | 57.6 | 2 | 52.9 | 65.4 | 51.4 | 2.4 | 44.2 | 60.4 | 60.7 | 0.4 | 56 | 67 | 62.2 | 1 | 57.8 | 69.3 | 64.1 | 0.2 | 60.3 | 69.8 | 68.4 | 0 | 65.4 | 72.9 |
| Tension-type headache | 64.3 | 0.4 | 58.3 | 72.5 | 61.6 | 0.4 | 55.1 | 69.8 | 70.3 | 0 | 65.2 | 76.3 | 68.9 | 0.2 | 63.2 | 76.5 | 68.9 | 0.1 | 64.4 | 75.3 | 74.6 | 0 | 71 | 80 |
| Testicular cancer | 55 | 2.4 | 37.1 | 69.6 | 52.1 | 3.7 | 35.7 | 66.5 | 69.3 | 0.1 | 64.9 | 75.9 | 65.5 | 0.6 | 58.9 | 75.5 | 67.1 | 0 | 61.9 | 74.4 | 72.9 | 0 | 69.2 | 79.7 |
| Thalassemias | 17.2 | 57 | 3 | 30.8 | 19.3 | 48.4 | 3.9 | 32.3 | 31.6 | 23.3 | 20.7 | 41.7 | 10 | 88.6 | 4.2 | 11.5 | 26 | 35 | 16.1 | 35.9 | 27.9 | 21.7 | 20.4 | 33.9 |
| Thalassemias trait | 59.1 | 7 | 54.6 | 70.5 | 55.6 | 7.7 | 49.8 | 67.7 | 67.8 | 1.5 | 63.3 | 75.2 | 65.8 | 2.2 | 60.4 | 75.2 | 66.2 | 1.1 | 61.2 | 74.4 | 72.7 | 0.5 | 68.5 | 79.2 |
| Thyroid cancer | 62.3 | 1 | 56.5 | 70.8 | 61.7 | 0.2 | 54.9 | 68.8 | 69.9 | 0 | 65 | 75.4 | 69.3 | 0 | 63.7 | 76.2 | 69.8 | 0 | 65.6 | 75.6 | 74.9 | 0 | 70.9 | 80.4 |
| Tracheal, bronchus, and lung cancer | 58.3 | 0.2 | 52.5 | 65.5 | 56 | 0.2 | 50 | 62.6 | 62.7 | 0 | 57.3 | 69.5 | 61.3 | 0.1 | 55 | 68.5 | 63.6 | 0 | 58.1 | 69.5 | 70.3 | 0 | 65.4 | 76.4 |
| Turner syndrome | 58.6 | 7 | 54.1 | 69.9 | 55.1 | 7.7 | 49.3 | 67.2 | 67 | 1.5 | 62.5 | 74.3 | 65.1 | 2.2 | 59.7 | 74.5 | 65.3 | 1.2 | 60.4 | 73.4 | 71.7 | 0.5 | 67.5 | 78.1 |
| Unintentional firearm injuries | 63.4 | 1.6 | 57.5 | 71.7 | 59.9 | 2.2 | 53.5 | 69 | 69.8 | 0.2 | 64.4 | 75.6 | 68.6 | 0.5 | 63.2 | 76.5 | 67.7 | 0.3 | 62.9 | 74.6 | 74.5 | 0.1 | 71 | 80 |
| Urinary tract infections | 63.6 | 1.2 | 57.5 | 71.8 | 60.8 | 1.1 | 54.3 | 69.1 | 70.4 | 0.1 | 65.2 | 76.3 | 68.4 | 0.4 | 62.4 | 75.8 | 70.1 | 0.1 | 65.8 | 75.9 | 74.3 | 0.1 | 71 | 80 |
| Urogenital congenital anomalies | 47.5 | 12.7 | 47.5 | 57.2 | 41 | 16.7 | 39.3 | 52.3 | 60.3 | 2.2 | 55.1 | 68.7 | 52.5 | 8.9 | 42.6 | 68.3 | 61.6 | 3.8 | 56.7 | 70.9 | 67.8 | 3.1 | 64.6 | 76.2 |
| Urolithiasis | 65.6 | 0.1 | 59.9 | 72.4 | 63 | 0.1 | 56.7 | 70.4 | 71.1 | 0 | 66.7 | 76.2 | 70.2 | 0.1 | 64.7 | 77.1 | 70.1 | 0 | 65.8 | 75.2 | 75.3 | 0 | 71.7 | 80.6 |
| Urticaria | 63.3 | 1.5 | 57.5 | 71.7 | 60.4 | 1.5 | 53.5 | 69 | 69.8 | 0.2 | 64.4 | 76.2 | 68.2 | 0.5 | 62.3 | 76.4 | 68.2 | 0.2 | 63.6 | 75.2 | 74.2 | 0.1 | 70.9 | 80 |
| Uterine cancer | 63.5 | 0.1 | 57.5 | 70.8 | 61.4 | 0.1 | 55.1 | 68.8 | 69.4 | 0 | 64.2 | 75.3 | 69.2 | 0 | 62.9 | 76.2 | 70.3 | 0 | 65.6 | 75 | 73.7 | 0 | 69.2 | 79.8 |
| Uterine fibroids | 64.6 | 0 | 58.8 | 72.1 | 61.6 | 0 | 55 | 69.4 | 69.3 | 0 | 64.2 | 75.4 | 68.6 | 0 | 62.2 | 76.1 | 67.7 | 0 | 62.7 | 74.9 | 73.2 | 0 | 69.2 | 79.6 |
| Vascular intestinal disorders | 57.3 | 5.3 | 50.7 | 68.7 | 57.7 | 1.8 | 50.1 | 67.5 | 66.8 | 0.1 | 61.1 | 73.4 | 65.2 | 1.1 | 59 | 74.1 | 69.3 | 0.1 | 65 | 75.1 | 74.1 | 0 | 70.7 | 79.3 |
| Venomous animal contact | 63.8 | 1.6 | 58.3 | 72.4 | 60.9 | 1.3 | 54.3 | 69.7 | 69.9 | 0.3 | 65.2 | 76.2 | 68.7 | 1.2 | 63.9 | 76.4 | 68.5 | 0.1 | 64.3 | 75.2 | 74.4 | 0 | 70.9 | 80 |
| Viral skin diseases | 63.3 | 1.2 | 57.5 | 71.8 | 60 | 1.4 | 53.5 | 69.1 | 69.4 | 0.3 | 64.4 | 75.6 | 67.6 | 0.6 | 61.6 | 75.8 | 67.8 | 0.3 | 62.9 | 74.6 | 73.7 | 0.1 | 70.2 | 79.4 |
| Coal workers pneumoconiosis | NA | NA | NA | NA | NA | NA | NA | NA | 69.9 | 0 | 66.4 | 74.8 | 66.4 | 0.1 | 62.6 | 72.3 | 65.7 | 0 | 61.1 | 71.3 | 72.4 | 0 | 69.2 | 76.7 |
