# Supplementary material for: Estimating Health Adjusted Age at Death (HAAD)
Source: PLoS One. 2020 Jul 14;15(7):e0235955. doi: 10.1371/journal.pone.0235955 (PMC7360045; doi:10.1371/journal.pone.0235955)
Supplement: Attachment — Submitted filename: Response to reviewers.docx [file pone.0235955.s002.docx]

Review Comments to the Author

Reviewer #1:

Major comments

*1. I am uneasy with the term ‘baseline health’ used to define both past and expected future health. ‘Baseline’ makes me think of the health status of an individual/population at a point in time, rather than also being ‘conditioned’ on future health. Is there another, better term available?*

Reply: We agree that this term is confusing, and now use “health status” throughout the paper.

*2. As iHALE is a distributional measure, reporting average iHALE does not seem insightful (except for when pointing out that two conditions can have similar average iHALE but differ when looking at the distribution, as the authors do). It would make sense to background the average measure in the text.*

Reply: This is a good point. We have changed the text (Abstract, last sentence of Results) to more frequently use metrics other than the average:

“Many illnesses, such as epilepsy, are associated with a higher health status in high-income settings (Q1 in Japan=59.1) than in low-income settings (Q1 in Ethiopia=26.3).”

Page 15, paragraph 2:

“The difference in Q1 for schizophrenia between the US and Japan was larger than the difference between the US and Ethiopia (Haiti 35.3, Ethiopia 35.6, China 39.3, Mexico 38.7, US 38.2, Japan 42.1). The same applies to the average HEAD.”

Also page 15, paragraph 2:

“Variability in Q1 for epilepsy between countries was high (Ethiopia 26.3, Haiti 30.5, Mexico 46.8, China 50.4, US 53.3, Japan 59.1). In addition, there is a much more unequal HEAD distribution for epilepsy in countries with a low Q1 (difference between quartiles (Q3-Q1) was 19.9 in Ethiopia, 17.9 in Haiti, 15.2 in China, 14.9 in Mexico, 13.3 in Japan, and 13.2 in the US).”

*3. More information on GBD sources are required, as characteristics - particularly the reliability of age-specific prevalence – would appear vital to the computations of iHALE. Are the illness data adjusted or modelled in any way, or are they taken directly from surveillance systems or routine recording? For some of the countries I am sceptical that data by 5-year age-group would have been available. Please consider the limitations of the GBD source.*

Reply: We agree that more information could be added to the paper. We now write (page 8, paragraph 1):

“Estimates for all countries and for all diseases in 2017 are obtained from the freely available GBD results tool [23]. The GBD Study produces cause-specific estimates of deaths, incidence, and prevalence by country, year, age, and sex, utilizing demographic methods [24, 25], ensemble models [26] using vital registration and verbal autopsy data about causes of death, and Bayesian meta-regression [27] using data from reviews of literature, registries, and hospitals.”

and we discuss several limitations of the input data on page 18, paragraph 1. We have added to that paragraph.

“Although estimates for 282 causes of death and 354 conditions from the GBD were available by five-year age groups and both sexes for 195 countries, these are based on sparse data in many low- and middle-income countries and rely heavily on modeled relationships with covariates and other data from the same region.”

Other comments

*1. Intro lines 19-20: the focus is described as how a particular illness affects total lifetime health. It is only future health that can be affected; please edit.*

Reply: We agree that the illness itself only affects future expected health. Differences in the distribution of age of onset of diseases also mean that past health differs across diseases. We have more explicitly described these two components of lifetime health in this section. The text now reads (second paragraph of Introduction, final sentence):

“Technically, only the future health (after disease onset) is affected directly by the disease, but lifetime health is the sum of past (before disease onset) and future health expectancies. These differences in years of healthy life lived before disease onset among people with different diseases also inform the potential lifetime health that can be attained and are part of measuring health status by the view we use.”

*2. I wonder about the assertion that iHALE ‘measures … health at an individual level’ (Abstract and elsewhere); in theory perhaps, but in practice (as demonstrated in the current paper), it is computed for strata of all individuals with a certain condition at a certain age. The authors may wish to re-think the use of ‘individual ’ and edit appropriately.*

Reply: We agree, and now use health expected age at death (HEAD) instead of iHALE. Further, the text has been changed as follows (Abstract, Methods, first sentence):

“HEAD measures health status for individuals with specific conditions, ...”

and (Methods, first sentence)

“HEAD measures lifetime health for individuals with specific conditions, …”

and (Discussion, third paragraph, 2^nd^ and 3^rd^ sentences)

“HEAD could be converted to a gap-measure by using the YLL method applied in GBD. Shortfall in life years could be calculated at disease onset by using the lowest mortality by age in the world as a reference.”

*3. The description of YLD as ‘in one particular year’ and ‘during the year’ is only relevant for the prevalence-based approach to computing YLD and DALY, such as used by the GBD project; please clarify.*

Reply: We have specified this in the text. (Introduction, 4^th^ paragraph, 3^rd^ sentence:

“GBD uses a prevalence-based approach, where DALYs are calculated for a set of diseases by summing the years of life lost (YLL) compared to a reference life expectancy and years lived with disability (YLD) in one particular year due to each disease”

*4. HALE needs to be defined at first mention.*

Reply: The HALE abbreviation is defined in the fourth paragraph in the Introduction. We have added another sentence to this paragraph that describes HALE:

“HALE measures the life expectancy in a population, adjusting for the disability experienced in the population, using age-specific mortality rates and YLDs per capita [22].”

*5. Methods line 8. If Ann can die in her 51st year, then it seems that she ‘will most certainly die within 21 years’ (not 20).*

Reply: We agree, and this has been corrected accordingly

*6. Methods/Data lines 9-10. Edit needed - for disease prevalence, the source must not only be the GBD ‘cause of death’ database.*

Reply: We have corrected this mistake, and now refer to “GBD results tool” (second to last sentence, first paragraph in *Data*). We also changed the reference to the GBD results tool website.

*7. Concl line 4: ‘concerned about giving higher priority’ is unfortunately ambiguous (ie. want to.. or want to avoid..)*

Reply: We agree, and have rephrased (first paragraph in Conclusion, second sentence): “Policy makers, supported by the ethical literature, may want to give higher priority to the worse off.”

*8. Concl line 10. ‘measure that is sensitive to distribution’. This is a bit awkward.. ‘distribution of what’? Please reword, given that iHALE is a distribution, and summary statistics (T20, quartiles etc) can be produced from it.*

Reply: We agree that this was vague, and the text now says (second to last sentence in Conclusions):

“Here we have presented a method for calculating HEAD, illustrated with examples how to estimate the distribution of health across individuals, and shown why considering these distributions is relevant for priority setting in health care and the measurement of population health.”

*9. Fig 2 : the word ‘age’ in the plot area is distracting and not described. Also suggest to change ‘Mean age’ to ‘Mean age of onset’*

Reply: We have removed “age” from the figure, and corrected “Mean age” as the reviewer suggested.

*10. Fig 3 is very hard to interpret. In the top panel, does ‘Death’ refer to the red area or to a point in time (on x-axis)? Should the rightmost endcap on PID length not be flush with the right border of the orange area? The text in the red area is illegible. Why do coloured areas slope upwards? Please improve and edit accompanying description.*

Reply: The figure text now reads (changes highlighted in bold):

“Outline of the conceptual structure of the **Health Expected Age at Death (HEAD)** method, where we calculate the sum of past health and expected future health for five individuals with different diseases (AML, ALL, epilepsy and schizophrenia). For a disease D, the dashed black line is background mortality, and the blue line is background mortality added to the excess risk of death caused by D. **The grey area is HEAD_D_.** The orange area is background health loss due to disability (dw), and the red area is health loss caused by D (dw_D_). **These areas slope upwards because disability increases with age.** The sum of the grey, orange and red areas constitute expected **age at death (EAD)**. The top solid black line gives a period after the onset of D when the person had a period of increased mortality (PIM). The bottom line gives a similar period of increased disability (PID). **Note that PIM and PID may be over before death occurs because the individual has survived the course of the disease, as seen in the top example, or PIM and PID may last beyond the death because the person died during the course of the disease, as seen in the other examples.**”

Reviewer #2:

*I do not agree that these are outcomes on the level of the individual, as these are measures for groups: groups with the same age and disease. So the i in iHALE to me is not justified.*

Reply: We agree, and now use HEAD instead of iHALE (see reply to Reviewer 1).

*Page 13 is confusing, as it is unclear what are the data that are used here. Only half way down the page I understood that this are data constructed from life table data, and this part is explaining the conceptual calculations behind the measure. An example of something that put me on the wrong foot was: “First, we define Y, the maximum age in our lifetable, by setting the chance of surviving from age Y to Y+1 to zero.” This is not a definition of Y, but a statement that one arbitrarily is assuming a particular Y. Reading this I also want to understand what value Y is used, which is only mentioned much later.*

Reply: We have edited the text as follows (Part I: Estimating disease-specific expected age at death, changes in bold):

“**To estimate expected age at death (EAD) from the GBD data described in previous sections, we use standard lifetable methodology, so that EAD** of an individual is simply

$\mathbf{EA}\mathbf{D}\left( \mathrm{age} \right)=age+\mathrm{LY}^{\mathrm{future}}(age)$ ,	(1)

where “age” is the age of the individual and LY^future^ is the number of life years **they have** left to live at their respective age. LY^future^ is not known until the individual dies, but its distribution can be estimated as follows. First, we **choose** Y, the maximum age in our lifetable, by setting the chance of surviving from age Y to Y+1 to zero. **In principle, Y can be any age, but we have used Y=99 throughout this paper.**”

*Equation (7) needs more explanation, as I can not grasp what is happening here; I seem to me that there is some summation over time missing.*

Reply: We have added the following text to clarify:

“…where *c* is current age and *d* is age at death. Note that it is only necessary to sum over time when *c<d*.”

*In table 1 it is not clear whether the event rates are rates or one-year probabilities.*

Reply: From GBD, prevalence, incidence, and YLDs are rates, not probabilities (the denominator is mid-year population estimates from GBD, which is used as the denominator for rates in GBD). Deaths from GBD were downloaded as rates for 5-year age groups for a single year. To do calculations for specific ages, we assumed the same rate for each single age in the age group. We treated these 1-year rates as probabilities since we’re adding rates to rates. Since we used rates (mx) and treated it as probabilities (qx), there could be small differences in results if anyone tries to replicate using qx from GBD.

*Also in table 1 it is unclear to me why dw is divided by 100000. YLD is normally given for a population. Is YLD in the formula the YLD for a population of 100000? Is this age dependent?*

Reply: The initial downloaded value is the rate of YLDs per 100,000. This is also true for prevalence and incidence, though we specified in the Table that those metrics are given per population. We have changed the description to reflect that YLDs are also given per population and changed the equations for dw and dwD accordingly. These rates are age-specific, which we have added to the formula. These are the new formulas:

$$dw(age)=\mathrm{YLD}_{All causes}(\mathrm{age})$$

$$\mathrm{dw}_{D}(age)=\frac{\mathrm{YLD}_{D}(age)}{P_{D}(age)}$$

*More explanation is needed, also for the formula used in the last row of the table.*

Reply: We have added detail on the last row of the table for the disability weight during the period of increased disability. The description now reads as follows:

“In the GBD study, disability weights are aggregated by subtracting from 1 the product of 1 minus the two disability weights. To calculate the disability weight during the period of increased disability during illness, we can combine the “background” disability from other causes on average and the disability specifically from the disease on average.

$dw_{D}^{\mathrm{PID}}(age)=1-\left( 1-\mathrm{dw}_{\mathrm{Background}}(age) \right)\left( 1-\mathrm{dw}_{D}(age) \right)$ .

To calculate the background disability, we treat YLD rates in the population as average disability weights for a given person and solve for the background disability by breaking down the all-cause disability weight into disability from the specific cause and from other causes.

$$\mathrm{YLD}_{All Causes}(age)=1-\left( 1-\mathrm{dw}_{\mathrm{Background}}(age) \right)\left( 1-\mathrm{YLD}_{D}(age) \right)$$

Solving for the background disability and inserting into the first equation, the disability weight during the period of increased disability is

$\mathrm{dw}_{D}^{\mathrm{PID}}(age)=1-\left( 1-\frac{\mathrm{YLD}_{All causes}(age)-\mathrm{YLD}_{D}(age)}{1-\mathrm{YLD}_{D}} \right)\left( 1-\mathrm{dw}_{D} \right)$ .

After the period, dw returns to that of the background population (dw).”
