# Supplementary material for: Estimating Health Adjusted Age at Death (HAAD)
Source: PLoS One. 2020 Jul 14;15(7):e0235955. doi: 10.1371/journal.pone.0235955 (PMC7360045; doi:10.1371/journal.pone.0235955)
Supplement: Attachment — Submitted filename: Response to reviewers2.docx [file pone.0235955.s003.docx]

**Response to reviewers, part II**

Dear editor

Please find our replies to the reviewers below.

**Reviewer #1**:

1. First, why (besides forming a convenient English-acronym) was the change to HEAD made? 'Health expected age at death' does not make sense to me. 'Expected age at death' does, including the provision (Eq 4) that the expected life years remaining is dependent on the disease that an individual has at age 'age'. The vital difference between EAD and the HEAD measure (if I understand correctly) is that in the latter life-years are adjusted for experienced disability/non perfect health; this is the main reason why 'age at death' is not an accurate descriptor. Please improve the name.
   a. We have now changed the name to Health Adjusted Age at Death (HAAD), and hope that this addresses the concern of the reviewer. Both “Life Expectancy” and “Age at Death” are measured in years, and these years can be health adjusted. In principle, HALE at birth is the same as expected HAAD at birth.
      We will also get back to the name of the metric in our reply to R2.
2. Related to this comment, T2 and other reported distribution-summaries refer to 'healthy life years' in the text when I guess they should have referred to 'HEAD; this is confusing.
   a. We agree, and now use HAAD where appropriate (twice in the final sentence in Methods section in the Abstract).
3. Second, what is the difference between 'health status' and 'lifetime health'? They seem to be used interchangeably throughout the paper. 'Health status at disease onset' should not include the 'future health' component (but is used - I think - when discussing HEAD, for instance 1st sentence of Conclusion, and in 3rd para of Introduction (it is not intuitive to me that young MS patients have a 'low health status').
   a. We think it is appropriate to use “health status” early in the Introduction, when we discuss the three perspectives current health, future health, and lifetime health. However, starting from the fourth paragraph of the Introduction, we now use “lifetime health” instead of “health status”.
   b. We have changed the last sentence in the third paragraph of the Introduction: “Conditions that are severe from a lifetime health perspective, like multiple sclerosis in young patients, do not necessarily require urgent interventions. To underscore that we use a lifetime health perspective when measuring health status, we will from now on use the term “lifetime health” instead of “health status”.”
4. Third, what do 'background mortality' and 'background disability' mean? Please explicitly define.
   a. We agree that this was confusing, and have made the following changes in the manuscript:
      i. We have introduced “baseline mortality” and “baseline disability” to separate between the mortality and disability among all people in the population (baseline) and mortality and disability among all people in the population without condition D (background).
         1. The first sentence below Eq. (5) now reads “In this section we will explain how to estimate HAA^past^ and $\mathrm{HALY}_{D}^{\mathrm{future}}$ using the baseline disability (i.e., the average disability in the population), dw, and the excess disease-specific disability, dw_D_ (Table 1).”
         2. The row explaining q in Table 1 now reads: “All cause probability of death in single-year intervals, for a total population (i.e., baseline mortality).”
         3. The legend and figure text of Figure 3 now use “baseline” instead of “background”, and the text includes the sentences:
            “For a disease D, the dashed black line is baseline mortality (i.e., average mortality in the population), …”
      ii. We use “background disability” and “background mortality” to describe the disability and mortality of those individuals in the population who do not have condition D.
          1. The row explaining $\mathrm{dw}_{D}^{\mathrm{PID}}$ now includes the sentence “To calculate the disability weight during the period of increased disability during illness, we can combine the “background” disability (i.e., disability from other causes than D) on average and the disability specifically from the disease on average.”

**Reviewer #2:**

5. The proposed metric now has a new name. Although this omits "individual", it still does not cover the content of the new concept, as the concept seems to be “expected healthy years lived until death”
   In this sense I would considered this to be simple a HALE, but calculated for a subgroup of the population (the group that is diagnosed with a particular disease at a certain age). Therefore I wonder if a new term is needed at all. I would suggest a name that reflects this is a type of HALE (e.g. HALE-AD ) (HALE by age of disease incidence).
   a. We have now changed the name to Health Adjusted Age at Death (HAAD). We do not agree that this is HALE for a subgroup of the population, because HALE considers future healthy life expectancy only. This also applies to LE (e.g., LE for a 99-year-old is about 1).

      Further, we do not agree that we should include “age of disease” or a similar phrase in the name, because one could calculate HAAD for individuals who are not affected by a condition (similar to what we did in Figure 1).

      Next, we have removed the word “expected/expectancy” from the name, because this obscures the concept of HAAD. For example, one could discuss the HAAD of disease D without knowing the expected HAAD of an individual or a group of individuals. Instead we use other words to describe the context in which HAAD is used, and introduce “aHAAD” as “average lifetime health/average HAAD”. See for example

Title:

1. “Estimating Health Adjusted Age at Death” instead of “Measuring Health Adjusted Age at Death”

Introduction:

2. “In this paper, we present a method for estimating HAAD, and show how to use the estimated HAAD to rank conditions at disease onset”

Methods:

3. “The diseases have distinct properties that highlight certain characteristics of HAAD.”
4. “We next set out to estimate the HAAD distribution in a population”
5. “In $\mathbb{H}_{D}^{\mathrm{HAAD}}$, row number *c* estimates HAAD for individuals who are *c* years old, whereas column number *d* estimates HAAD for individuals who will die at age *d*.”
6. “As opposed to the discrete AD, HAAD is continuous. For example, one individual who dies at age 80 may have HAAD=67.3, whereas another could have achieved 67.4 or 67.5.”
7. “Because we do not have access to data on individuals, every individual with the same condition and the same age of onset is assumed to have the same HAAD distribution.”
8. “See Figure A1 (Appendix) for details on incidence assumptions that are being used in HAAD calculations for AML and ALL in the US.”

Results:

9. “Table 2 shows estimated aHAAD, T20, Q1 and Q3 for ALL, AML, schizophrenia and epilepsy in six countries”

Discussion:

10. “We show how two conditions, ALL and AML, with similar estimated aHAADs have substantially different HAAD distributions.”
11. “The relevance of HAAD is particularly good for preventive interventions for a disease where you are likely to capture benefits across a range of ages”
12. “In this article we present HAAD as an achievement measure”
13. “Understanding the underlying reasons for differences in HAAD distributions can have policy implications.”
14. “It is important to note that as a measure of lifetime health among people with a specific condition, HAAD is influenced by mortality risk and morbidity from other causes, as well as by the age at which the disease occurs”

6. One big problem with this paper is that it seems to use the terms YLD and dw at some places as a kind of synonyms, while these are quit different concepts.The authors should fix this problem with the paper, as this prevents me from fully understanding their calculations.

   Some examples of this:

   Table 1:

   dw(age) = YLDAll causes(age)

   How can this be? YLD is a number for the population, which will be much larger then 1, while dw is between 0 and 1.
   Because of this I can also no follow the next part in the last row of the table:

   YLD.All Causes(age) = 1 − (1 − dwBackground(age))(1 − YLDD(age))

   Again, 1 - YLD does not make sense to me, as YLD usually is much larger than 1.


   Similarly, in the text of the introduction: ”HALE measures the life expectancy in a population, adjusting for the disability experienced in the population, using age-specific mortality rates and YLDs per capit”. HALE does not use YLDs but dw’s.
   a. Reply: We understand that this was confusing. GBD calculates YLDs by summing dw’s over all individuals in the population. Hence,

      average dw = YLD/(population size) .

      In other words, dw is *per capita* YLD, and

      and

      average dw for disease D = (YLD caused by disease D)/(population with disease D) .
   b. Changes: We have changed the text in Table 1 and emphasized on several occasions that YLD is *per capita*.
      i. Discussion:
         “Shortfall in disabilities could use the lowest YLD rates (i.e., YLD *per capita*) across countries as a reference for disability shortfall.”
      ii. Table 1
          “Note that $\mathrm{YLD}_{All causes}(\mathrm{age})$ is *per capita*.”
          “Note that $\mathrm{YLD}_{D}(\mathrm{age})$ is *per capita*.”
          “To calculate the background disability, we treat YLD rates (i.e., YLD *per capita*) in the population as average disability weights for a given person and solve for the background disability by breaking down the all-cause disability weight into disability from the specific cause and from other causes.”
          “Note that $\mathrm{YLD}_{All causes}(\mathrm{age})$ and $\mathrm{YLD}_{D}(\mathrm{age})$ are *per capita*.”
7. The calculations treat rates as probabilities. This will work reasonably as long as rates are low. However, they will no longer be so at high ages. Why do you not simple use prob =1- exp(-rate*interval length) (assuming a constant rate in the interval) to calculate the probability?
   a. As suggested, we have changed all of our calculations using q = 1-exp(-M), where M is the mortality rate and q is the probability of dying during a year. The interval length is 1 in all our calculations.
   b. We have also clarified the difference between mx and qx in Table 1:

      “*All cause probability of death in single-year intervals, for a total population (i.e., baseline mortality).*
      From GBD 2017.

$$q=1-\exp\left( -M_{All causes} \right)$$

Converted from single-year M_D_ using common demographic approximation [32].”

and

“Probability of death due to disease and baseline mortality. These are not given directly in GBD, but can be calculated using

$q_{D}\left( \mathrm{age} \right)=1-\exp\left( -\left( M_{All causes}\left( \mathrm{age} \right)-M_{D}\left( \mathrm{age} \right)+em_{D}\left( \mathrm{age} \right) \right) \right)$	.

Substituting em_D_ into q_D_ yields

$q_{D}\left( \mathrm{age} \right)=1-\exp\left( -\left( M_{All causes}\left( \mathrm{age} \right)+M_{D}\left( \mathrm{age} \right)\left( \frac{1}{P_{D}\left( \mathrm{age} \right)}-1 \right) \right) \right)$	.

We can see that if P_D_=1, meaning that all individuals in the population have a disease, q_D_ simply becomes q. This is also the case if there is no mortality from disease, so that M_D_=0.”

8. Methods, line 13: The use of “health status” here is confusing, as the example only refers to being alive.
   a. We have changed the relevant text to:
      “For simplicity in this example, although we will use the term “lifetime health”, we disregard health/disability adjustment for time with illness and focus only on their age at death. Because there is a 99% probability that Ann will die at a younger age than Bob will, Ann’s lifetime health is lower than Bob’s in terms of total length of life (past life plus expected future life), even though Bob’s expected future life is shorter.”
9. Methods, line 21: Here it is claimed that HEAD also looks at distributions, while in the example only the average (=expectancy) is used. I therefore could not follow the reasoning in this part.
   a. We have changed the text to:
      “HAAD enables comparison of both average lifetime health (aHAAD) and distribution of lifetime health between individuals with different diseases (e.g., at disease onset, Ann’s disease will have a very different HAAD distribution than Bob’s disease).”
10. At parts the text still pertains that the metric is for individuals (while the metric is for groups of individuals). For instance in the conclusion:
    “Here we have presented a method for calculating HEAD, illustrated with examples how to estimate the distribution of health across individuals”
    a. In principle, HAAD could be used at an individual level, but that would require information about disease history and age at death. However, as the reviewer correctly points out, the aim of this paper is to estimate HAAD for groups of individuals. Still, we focus on the estimated HAAD distribution across those individuals.
    b. We have changed the relevant text:
       “Here we have presented a method for estimating lifetime health by considering HAAD, illustrated with examples how to estimate the distribution of HAAD across individuals, and shown why considering these distributions is relevant for priority setting in health care and the measurement of population health.”
11. In the legend of figure 3 it is strange that PID is shorter than PIM. Although this is just a legend, it confuses.
    a. We have changed Fig 3 so that PID is now longer than PIM.

Because we changed the calculations (as suggested) by using q = 1-exp(-M), the figures and tables changed. We have also edited the manuscript for clarity.

Best wishes,

On behalf of the authors,

Øystein A. Haaland
