# Supplementary material for: Estimating Health Adjusted Age at Death (HAAD)
Source: PLoS One. 2020 Jul 14;15(7):e0235955. doi: 10.1371/journal.pone.0235955 (PMC7360045; doi:10.1371/journal.pone.0235955)
Supplement: Attachment — Submitted filename: Response to reviewers3.docx [file pone.0235955.s004.docx]

**Response to reviewers, part III**

Dear editor

Please find our replies to the reviewers below.

**Reviewer #1**:

Comment:

“The authors have achieved a lot with respect to clarification and use of terminology since the previous revision.

Main remaining point to be addressed:
The use of disability weight (dw) to stand for disability as a quantity with a time dimension will be very confusing to the reader familiar with 'dw' as an elicited weight (not something that can be seen as a population average, or that necessarily can be decomposed).

In Table 1, the authors describe the calculation of "... the background disability, we treat YLD rates (i.e., YLD per capita) in the population as average disability weights for a given person and solve for the background disability by breaking down the all-cause disability weight into disability from the specific cause and from other causes.

I would ask the authors to not conflate disability weight and YLD rate, and so choose terms that do not overlap with 'standard' burden of disease terminology.”

Reply:

Although we understand the concern of the reviewer, according to the GBD, “YLDs were estimated as the product of prevalence estimate and a disability weight for health states […]” (Lancet 2018; 392: 1789–858). In Table 1 this corresponds to:

$\mathrm{dw}_{D}(age)=\frac{\mathrm{YLD}_{D}(age)}{P_{D}(age)}$ ,

and the rest of the derivations follow from this. This terminology may be confusing, but we worry that it will be even more confusing if we introduce new terminology do describe the relationship between YLDs and disability weights. Therefore, we prefer to keep the terms YLD and “disability weight” as they are.

To explain the GBD approach, the Introduction says:

“GBD uses a prevalence-based approach, where DALYs are calculated for a set of diseases by summing the years of life lost (YLL) compared to a reference life expectancy and years lived with disability (YLD) in one particular year due to each disease [19]. For a particular condition and a particular year, YLL is the sum of all the years lost for the individuals who died from the condition during that year. The reference is the age-adjusted life expectancy (LE) from a life table derived from the mortality rates in the locations with the lowest age-specific mortality in the GBD study [20]. YLD, on the other hand, is the sum of the health loss due to the condition during the year across people living with the condition [21]. DALYs aggregated from YLLs and YLDs are a measure of overall population burden. HALE measures the life expectancy in a population, adjusting for the disability experienced in the population, using age-specific mortality rates and YLDs *per capita* [22].”

In Table 1 the definition of YLD_D now reads:

“*Years Lived with Disability (per capita in one year) of disease in 5-year age intervals.*

From GBD 2017.

Assumed to be the same in all 1-year intervals.”

We hope that you find this line of reasoning convincing.

Best wishes,

On behalf of the authors,

Øystein A. Haaland
